# Two-Step Engineering of Food-Grade *Aspergillus oryzae* via Endogenous Signal Peptides and Vesicle Trafficking Proteins to Enhance Carrier-Free Protein Secretion

**DOI:** 10.3390/jof12040289

**Published:** 2026-04-18

**Authors:** Sarocha Panchanawaporn, Nakul Rattanaphan, Sukanya Jeennor, Jutamas Anantayanon, Weerapong Woraprayote, Laphaslada Pumpuang, Thipphiya Karirat, Nuttamon Prompakdee, Kobkul Laoteng, Chanikul Chutrakul

**Affiliations:** 1 Industrial Bioprocess Technology Research Team (IIBT), Functional Ingredients and Food Innovation Research Group (IFIG), National Center for Genetic Engineering and Biotechnology (BIOTEC), National Science and Technology Development Agency (NSTDA), Thailand Science Park, Phahonyothin Road, Khlong Nueng, Khlong Luang, Pathum Thani 12120, Thailand; sarocha.pan@biotec.or.th (S.P.); nakul.rat@biotec.or.th (N.R.); sukanya.jee@biotec.or.th (S.J.); jutamas.ana@biotec.or.th (J.A.); thipphiya020@gmail.com (T.K.); nut57nuttamon@gmail.com (N.P.); kobkul@biotec.or.th (K.L.); 2 Food Biotechnology Research Team, Functional Ingredients and Food Innovation Research Group (IFIG), National Center for Genetic Engineering and Biotechnology (BIOTEC), National Science and Technology Development Agency (NSTDA), Thailand Science Park, Phahonyothin Road, Khlong Nueng, Khlong Luang, Pathum Thani 12120, Thailand; weerapong.wor@mahidol.ac.th (W.W.); laphaslada.pum@biotec.or.th (L.P.)

**Keywords:** *Aspergillus oryzae*, secretory system, protein secretion, signal peptide, Sec1/Munc18 protein, vesicle trafficking, fungal strain improvement, fungal biofactories

## Abstract

Heterologous protein secretion in filamentous fungi is often constrained by limitations in signal peptide recognition and intracellular trafficking. *Aspergillus oryzae*, a food-grade industrial fungus, has a robust native secretory system. However, its capacity for recombinant protein secretion remains suboptimal. Here, we developed a two-step, carrier-free engineering strategy to enhance protein secretion in *A. oryzae*. We identified endogenous signal peptides among highly secreted proteins using a green fluorescent protein (GFP) reporter. The oryzin signal peptide SPAoalp1 increased GFP secretion 5.50-fold compared with a no-signal-peptide control. We co-overexpressed *Aosly1*, a Sec1/Munc18 family protein that regulates soluble N-ethylmaleimide-sensitive factor attachment protein receptor–mediated vesicle trafficking, which, in combination with *SPAoalp1*, increased secretion approximately two-fold compared with SPAlp1 control and ten-fold with no-SP control. Applying the engineered platform for genetic improvement of heterologous bovine κ-casein increased secretion from 0.11 to 0.24 mg/L. Physiological optimization further increased secretion. The developed system provided initial evidence for secretion of a ~12 kDa band consistent with *Aopafb* transcription, with MIC_90_ values of 4.56–8.24% (*v*/*v*) against two *Candida albicans* strains and 4.68% (*v*/*v*) against *Aspergillus niger*. The system offers a modular framework for engineering fungal secretion and expands the utility of *A. oryzae* for recombinant protein production.

## 1. Introduction

Filamentous fungi have a long history of use in food production. Commonly used filamentous fungi include *Monascus* spp., *Penicillium* spp., *Rhizopus* spp., and *Fusarium venenatum*, which are used to produce red yeast rice, ripened cheese, fermented tempeh, and mycoprotein, respectively [1,2,3,4]. Some *Aspergillus* spp. have been widely exploited in food and industrial biotechnology. Commonly used *Aspergillus* species include *Aspergillus niger* and *Aspergillus oryzae.* Both of these species are considered Generally Recognized as Safe (GRAS) organisms [5,6,7,8]. *A niger* has been widely used for the production of industrial enzymes and organic acids [9]. With a long history in traditional Asian fermentation, *A. oryzae* can be safely used to produce numerous proteins, enzymes, organic acids, and specialized metabolites of industrial value. As part of the industrial *Aspergillus* production platform, strains of *A. oryzae* used in industrial food processing are strictly considered non-aflatoxigenic, as determined by inactive or non-functional aflatoxin biosynthetic gene clusters [10,11]. The ability to grow on inexpensive substrates and secrete large quantities of hydrolytic enzymes and secondary metabolites makes this fungus a robust microbial platform [12]. Beyond native hydrolytic enzymes, *A. oryzae* can also produce structurally complex functional proteins. Based on various genetic and cellular engineering strategies, heterologous protein production in filamentous fungi has been extensively reviewed [13]. *A. oryzae* is particularly suited for recombinant protein expression because it can perform complex eukaryotic post-translational modifications (PTMs) via the secretory pathway [14]. Recent advances in precision gene editing, including CRISPR-Cas9–based tools and native strong promoters, have further expanded the potential for strain improvement [15,16,17,18].

Protein secretion in filamentous fungi relies on coordinated secretory pathway processes [19]. Nascent polypeptides are co-translationally delivered to the endoplasmic reticulum (ER) via signal peptides (SPs) and signal recognition particles. Within the ER, folding, glycosylation, and phosphorylation by resident chaperones and enzymes ensure protein stability and trafficking competence [20]. Vesicles then transport proteins to the Golgi apparatus (GA) and then to the extracellular space via exocytosis. These processes are controlled by Rab GTPases and soluble N-ethylmaleimide-sensitive factor attachment protein receptor (SNARE) proteins, including vesicle and target SNAREs that mediate membrane fusion [21]. Sec1/mammalian Unc-18 (Sec1/Munc18 or SM) proteins such as Sec1 and Sly1 regulate SNARE complex assembly and membrane fusion efficiency. Therefore, they are critical for protein secretion [21,22,23]. Although *A. oryzae* has a naturally robust secretory system, heterologous protein secretion is often inefficient and unstable. Previous strategies to improve secretion in *A. oryzae* include fusion of target proteins to amylase SPs [24], co-expression of carrier fusion proteins combined with disruption of degradative pathways [25], and co-expression with folding regulators such as HacA [26]. However, these approaches typically rely on carrier fusion partners, complicating downstream purification with the potential to alter the properties of the target proteins.

**Figure 1:**
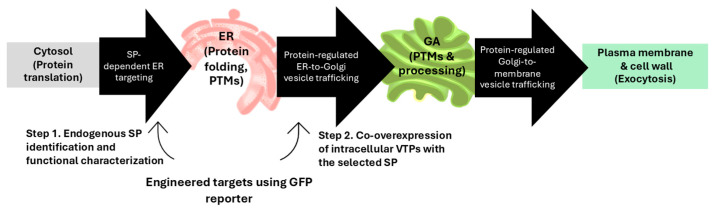
Schematic of the filamentous fungal secretory pathway and two-step engineering targets leveraged in this study to enhance carrier-free protein secretion. Abbreviations: GFP, green fluorescent protein; SP, signal peptide; ER, endoplasmic reticulum; PTMs, post-translational modifications; GA, Golgi apparatus; VTPs, vesicle trafficking proteins.

To address these limitations, we aimed to strategically enhance the secretory capacity of *A. oryzae* using a genetic approach, without relying on carrier fusion proteins. A schematic of the filamentous fungal secretory pathway and the two-step engineering targets for enhancing carrier-free protein secretion is shown in Figure 1. We used an auxotrophic, morphologically optimized and low-protease strain [27] as the host and performed a two-step workflow. First, we identified and functionally characterized endogenous SPs among highly secreted proteins using a green fluorescent protein (GFP) reporter. Second, intracellular vesicle trafficking proteins (VTPs), which were used as regulatory components, were co-overexpressed and evaluated on SP-targeted protein cargos. These had already entered the ER to further improve secretion. We validated the engineered system by testing the secretion of bovine κ-casein. In addition, this provided initial evidence for the production of active culture supernatant of the constructed AoPAFB-expressing strain. κ-casein is a major component of bovine milk proteins. It belongs to the casein family that constitutes approximately 80% of total milk protein and comprises 169 amino acids. This protein is applied in cheese production, dairy processing, and bioactive peptide generation [28,29,30]. PAFB derived from *Penicillium chrysogenum* contains 58 amino acids with a molecular weight of 6.5 kDa and three disulfide bonds. It is cationic and has an amphipathic structure. PAFB is one of the best-characterized anti-fungal proteins derived from filamentous ascomycetes in terms of structural organization, anti-yeast and fungal pathogen activities, and regulation of expression and production [31,32,33,34,35,36]. Using these two different-sized, structurally distinct proteins derived from phylogenetically diverse donors allows the evaluation of the engineered secretory system in *A. oryzae*. While κ-casein is a medium-sized phosphoprotein that requires eukaryotic PTMs, including glycosylation and phosphorylation [37], the compact PAFB structure relies on correct folding through disulfide bond formation [34]. Both proteins can be used to assess multiple aspects of secretion efficiency. The secretion can be achieved without carrier fusion, and co-overexpression of an intracellular trafficking protein provides a substantial additional enhancement. Our study has shown that combining an endogenous signal peptide with overexpression of a Sec1/Munc18 family regulator enables carrier-free, enhanced secretion of structurally diverse heterologous proteins in *A. oryzae*. The findings provide deeper insights into the mechanistic determinants of secretion efficiency in filamentous fungi and establish a broadly applicable framework for fungal secretory pathway engineering. The established system and modified host help improve fungal cell factories for producing proteins or peptides relevant to food industrial applications without the need for carrier fusions.

## 2. Materials and Methods

### 2.1. Microbial Strains and Cultivation

A previously constructed auxotrophic, morphologically optimized and low-protease strain of *A. oryzae* BCC7051 (Δ*pyrG*, Δ*ligD*, Δ*ags1*, Δ*prtR*) [27] was used as the recipient strain for all the transformations. The strain was maintained on Czapek Dox (CD) agar supplemented with 0.5% (*w*/*v*) uridine and 0.2% (*w*/*v*) uracil. For spore preparation, the fungus was cultured on rice medium at 30 °C for 5–7 d. The spores were harvested using 0.01% (*v*/*v*) Tween 80, and suspensions were adjusted to 2 × 10^6^ spores/mL before inoculation. For the liquid cultivation, 50 mL of basal semi-synthetic medium [(per liter): 40 g (*w*/*v*) of glucose, 0.2 g (*w*/*v*) of NH_4_Cl, 5 g (*w*/*v*) of yeast extract, 2.4 g (*w*/*v*) of KH_2_PO_4_, 0.5 g (*w*/*v*) of MgSO_4_∙7H_2_O, 0.1 g (*w*/*v*) of CaCl∙2H_2_O, 15 mg (*w*/*v*) of FeCl_3_∙7H_2_O, 10 mg (*w*/*v*) of MnSO_4_∙H_2_O, and 7.5 mg (*w*/*v*) of ZnSO_4_∙7H_2_O)] [27] or other specified media were dispensed into 250-mL Erlenmeyer flasks. They were then inoculated with the spore suspension and incubated at 30 °C on a rotary shaker at 200 rpm for 3 d.

*Saccharomyces cerevisiae* INVSc1 (*MATα his3-Δ1 leu2 trp1-289 ura3-52*; Invitrogen, Carlsbad, IL, USA) was used as the DNA assembly host for plasmid construction, following Pahirulzaman et al. [38]. Yeast cultures were grown in yeast extract–peptone–dextrose medium broth (1% yeast extract, 2% peptone, 2% glucose) at 30 °C with shaking at 200 rpm. Transformants were selected on synthetic dextrose medium containing 0.67% yeast nitrogen base without amino acids and 2% glucose, supplemented with L-tryptophan (0.002%), L-histidine (0.002%), and L-leucine (0.003%).

*Escherichia coli* DH5α (*supE44*, *ΔlacU169*, *(Φ80lacZΔM15)*, *hsdR17*, *recA1*, *endA1*, *gyrA96*, *thi1*, *relA1*; Thermo Fisher Scientific, Waltham, MA, USA) was used for propagation of recombinant plasmids. Transformants were grown in Luria–Bertani medium supplemented with ampicillin (0.01%, *w*/*v*) at 37 °C with shaking at 200 rpm.

### 2.2. Identification of Signal Peptide Sequences and Vesicle Trafficking Proteins

Putative genes encoding secreted proteins and intracellular trafficking factors in *A. oryzae* BCC7051 (reference genome/proteome dataset; GenBank accession OOO09832.1) [10] were identified using Basic Local Alignment Search Tool (BLAST) (BLAST+ v2.16.0) against the NCBI non-redundant nucleotide and protein databases (http://www.ncbi.nlm.nih.gov, accessed on 30 June 2024), with gene sequences from *A. oryzae* RIB40 or *S. cerevisiae* as queries. Accession IDs for each secreted protein with signal peptide are CAA25219.1, OOO09972.1, OOO09458.1, OOO12167.1, OOO13350.1 and OOO13707.1 for SPAnglaA, SPAoalp1, SPAoxynB, SPAofaeB2, SPAomreA and SPAopep, respectively. The vesicle trafficking protein analyzed are OOO11641.1, OOO07523.1, OOO06293.1 and OOO07634.1 for Aobet1, Aosso1, Aosly1 and Aosec1, respectively. SPs and predicted signal peptidase I cleavage sites were analyzed using SignalP (v6.0) [39] (https://services.healthtech.dtu.dk/services/SignalP-6.0, accessed on 10 July 2024). Transmembrane topologies of SNARE and SM proteins from *A. oryzae* BCC7051 were predicted using DeepTMHMM (v1.0) [40] (https://dtu.biolib.com/DeepTMHMM, accessed on 1 August 2024).

### 2.3. Plasmid Construction and Generation of Recombinant Strains

Expression plasmids containing individual SPs fused to the monomeric GFP reporter gene (*mgfp*) were constructed under the control of the constitutive glyceraldehyde 3-phosphate dehydrogenase (*gpdA1*) promoter (PAogpdA1) [18] (Figure S1). Homologous DNA fragments were assembled into a linearized backbone carrying the *AopyrG* selectable marker using yeast assembly [38]. SP–*mgfp* fusion fragments were amplified via Platinum Taq DNA polymerase (Invitrogen) from the *pPNGB* plasmid template [41] using gene-specific primers (Table S1). Constructs were recovered from *S. cerevisiae*, propagated in *E. coli* DH5α, and verified using DNA sequencing.

For VTP overexpression, SNARE and SM genes were cloned under the ubiquitin promoter (PAoubi) [18] using the oryzin signal peptide (SPAoalp1)–mgfp plasmid as a backbone (Figure S2). SNARE and SM coding sequences were obtained by reverse transcription polymerase chain reaction (RT-PCR) from *A. oryzae* BCC7051 total RNA using SuperScript III One-Step RT-PCR and appropriate primer pairs (Table S1).

To evaluate heterologous protein secretion, a series of constructs expressing codon-optimized *Aocsn3* (κ-casein) containing the native signal peptide were generated under the PAogpdA1 promoter: (i) *Aocsn3* (pAocsn3), (ii) *Aocsn3* fused to *SPAoalp1* (pSPAoalp1–Aocsn3), and (iii) co-overexpression of *SPAoalp1–Aocsn3* with *Aosly1* under PAoubi (pSPAoalp1–Aocsn3 + Aosly1; Figure S3A–C). For antifungal protein expression, the coding sequence harboring the mature PAFB protein with the native signal peptide (*AopafB*) was codon-optimized for *A. oryzae.* It was then fused in-frame downstream of *SPAoalp1*, generating pSPAoalp1–AopafB + Aosly1 (Figure S3D). All the constructs contained 5′ and 3′ homologous regions targeting integration into the *pyrG* locus of the *A. oryzae* host genome. The Gene^TM^ algorithm (GenScript, Piscataway, NJ, USA) was used for *A. oryzae* codon optimization. This was to optimize the codon adaptation index, balance the guanine and cytosine content, remove problematic sequence motifs such as internal restriction sites, sequence repeats, and cryptic splice sites, and avoid the formation of secondary structures in single-stranded RNA. Plasmids were introduced into the Δ*pyrG A. oryzae* BCC7051 strain using protoplast-mediated transformation [42]. Transformants were selected on CD agar lacking uridine and uracil, and correct genomic integration was confirmed using diagnostic polymerase chain reaction (PCR). Stable isolates were obtained following spore purification and repeated subculturing.

### 2.4. Quantification of GFP Secretion

GFP secretion was assessed using six independent transformants per construct. Culture supernatants were collected after growth in basal semi-synthetic medium by filtration through Miracloth (Merck Millipore, Darmstadt, Germany) under gentle suction to remove the mycelia. Aliquots (100 µL) of clarified supernatants were transferred into 96-well microplates. Fluorescence intensity was measured using a microplate reader with an excitation wavelength of 485 nm and an emission wavelength of 535 nm. Fluorescence values were recorded as arbitrary units (AU) and reported as the mean ± standard deviation (SD). GFP without a signal peptide was not expected to be actively secreted. The fluorescence detected in the wild-type and no-SP culture supernatants was treated as background, that is, culture matrix autofluorescence or trace release of intracellular components. The secretion efficiency was evaluated as a fold increase relative to the no-SP control or SPAnglaA, as appropriate.

### 2.5. Gene Expression Analysis by RT-PCR

Total RNA was extracted using the PureLink RNA Mini Kit (Thermo Fisher Scientific, Waltham, MA, USA), followed by DNase I treatment for 30 min to eliminate genomic DNA contamination. Expression of *gfp* and additional target genes was analyzed using the SuperScript III One-Step RT-PCR kit (Thermo Fisher Scientific). The gene-specific primers are listed in Table S2. *18S rRNA* was amplified as a reference gene. Amplified products were separated and visualized using agarose gel electrophoresis.

### 2.6. Analysis of Recombinant Proteins

#### 2.6.1. Preparation of Crude Protein Extracts

Culture supernatants were separated from the mycelia by filtration and further clarified through 0.2-µm membrane filters. Culture filtrates were concentrated 25-fold using centrifugal ultrafiltration devices (Amicon^®^ Ultra; 3-kDa molecular-weight cutoffs; Merck Millipore, Darmstadt, Germany). For intracellular protein extraction, 200 mg of freshly harvested mycelium was ground in liquid nitrogen and resuspended in 1 mL of 50 mM phosphate buffer (pH 7.4). Cell debris was removed by centrifugation at 13,780× *g* for 15 min at 4 °C. The resulting supernatant was collected as crude intracellular protein.

#### 2.6.2. Total Protein Quantification

A Bradford assay (Bio-Rad, Hercules, CA, USA) was used in the secretion experiments for total protein estimation and sodium dodecyl sulfate–polyacrylamide gel electrophoresis (SDS-PAGE) loading normalization. Samples were appropriately diluted, and absorbance was recorded at 595 nm. Bovine serum albumin standards (0.0375–0.5000 mg/mL) were used to generate a calibration curve.

#### 2.6.3. Sodium Dodecyl Sulfate–Polyacrylamide Gel Electrophoresis Analysis

Protein samples were separated by SDS-PAGE using polyacrylamide gels. This consisted of 5–7% stacking and 12–16% resolving layers in a Mini-PROTEAN^®^ Tetra Cell system (Bio-Rad Laboratories, Hercules, CA, USA). Samples were mixed with 4× Laemmli buffer (Bio-Rad), heated at 95 °C for 5 min and centrifuged. They were then loaded alongside commercial κ-casein (C0406; Sigma-Aldrich, St. Louis, MO, USA) or synthetic PAFB protein without a pre-pro signal peptide sequence (GenScript). Electrophoresis was conducted at 100 V for 15 min followed by 150 V for 1 h in 1× Tris–glycine–SDS running buffer. Precision Plus Protein^™^ prestained molecular-weight markers (Bio-Rad Laboratories) were used as references. Gels were stained with Coomassie Nano Blue (Bio-Helix, New Taipei City, Taiwan) and destained with distilled water under gentle agitation until protein bands were clearly visible. Gel images were captured using a gel documentation system. Analysis of PAFB and extracellular protein bands from the AoPAFB-expressing strain was performed using a Tris-tricine-SDS buffer system with 7% stacking and 16% resolving gels. Samples were prepared under strong reducing conditions by adding 100 mM freshly prepared dithiothreitol to the Laemmli dye and conducting extended heating for 10 min.

#### 2.6.4. Western Blotting

Aoκ-casein samples were separated on 12% SDS-PAGE gels using a Mini-PROTEAN electrophoresis apparatus (Bio-Rad Laboratories). Proteins were transferred onto 0.2-μm nitrocellulose membranes using a Mini Trans-Blot system (Bio-Rad Laboratories). Membranes were blocked with OneStep Blocker (Bio-Helix) and incubated for 1 h with bovine κ-casein polyclonal antibody (Bioss Inc., Woburn, MA, USA; 1:1000 dilution). This was followed by incubation with horseradish peroxidase (HRP)–conjugated anti-rabbit IgG (Cell Signaling Technology, Danvers, MA, USA; 1:1000 dilution). After washing, membranes were treated with Clarity^™^ Western enhanced chemiluminescence substrate (Bio-Rad Laboratories) for 5 min in the dark. Chemiluminescent signals were visualized using a ChemiDoc XRS imaging system (Bio-Rad Laboratories).

#### 2.6.5. Quantification of Recombinant Aoκ-Casein Using an Enzyme-Linked Immunosorbent Assay

Recombinant κ-casein titers were quantified with an enzyme-linked immunosorbent assay (ELISA) using a bovine κ-casein standard curve (0.188–1.000 μg/mL). Serially diluted culture supernatants with 100 µL per well were added to 96-well Immunoplates in triplicate and incubated for 16 h at 4 °C. After washing and blocking with BlockPro™ (Visual Protein, Taipei, Taiwan), the wells were incubated with bovine κ-casein polyclonal antibody (Bioss Inc., Woburn, MA, USA; 1:3500 dilution). This was followed by incubation with HRP-conjugated anti-rabbit IgG (Cell Signaling Technology, Danvers, MA, USA; 1:5000 dilution). 3,3′,5,5′-Tetramethylbenzidine substrate (Bio-Rad Laboratories) was added, and absorbance was measured at 450 nm.

### 2.7. Antifungal Activity Assays Using Broth Microdilution

#### 2.7.1. Anti-*Candida albicans* Assay

Antifungal activity against pathogenic yeasts was evaluated using *C. albicans* ATCC 10231 and ATCC 90028. Cultures were grown in tryptic soy broth (Thermo Fisher Scientific) at 30 °C for 24 h and adjusted to a final cell density of 2 × 10^5^ cells/mL. Synthetic PAFB protein was dissolved in sterile distilled water and two-fold serially diluted to final concentrations ranging from 0.8 to 50.0 µg/mL. Culture supernatants concentrated 25-fold using centrifugal ultrafiltration devices were further diluted to final test concentrations of 50, 25, 12.5, 6.25, 3.13, and 1.56% (*v*/*v*) for antifungal testing. Broth microdilution assays were performed in sterile 96-well plates using the modified Clinical and Laboratory Standards Institute M07 protocol [43]. Each well contained 100 µL of test sample and 100 µL of yeast suspension. This yielded a final inoculum of 2 × 10^4^ cells/well with a final cell density of 1 × 10^5^ cells/mL in 200 µL, enabling accurate colony counting on the plate. Amphotericin B (0.10–6.25 µg/mL) prepared in 5% dimethyl sulfoxide was used as the positive control. After incubation at 30 °C for 24 h, the solution in each well was mixed and serially diluted. The dilutions (100 µL) were plated on solid agar medium. After 24 h of incubation, viable cells were enumerated by plate counting to obtain colony-forming unit-based endpoints. Growth inhibition was calculated relative to untreated controls, and the minimum inhibitory concentration values causing ≥90% growth inhibition (MIC_90_) were determined.

#### 2.7.2. Anti-*Aspergillus niger* and Self-Inhibition Assays

Antifungal activity against a filamentous fungus was evaluated using *A. niger* DMST 15538. Spore suspensions were prepared in potato dextrose broth (Thermo Fisher Scientific) at a density of 2 × 10^4^ spores/mL. Test samples with synthetic PAFB protein or concentrated culture supernatants at the same dilutions were prepared as described in Section 2.7.1. Microdilution assays were conducted using the modified National Committee for Clinical Laboratory Standards M27-A2 protocol [44]. Each well contained a test sample and a spore suspension to achieve a final inoculum of 2 × 10^3^ spores/well (final spore density 1 × 10^4^ spores/mL in 200 µL). This facilitated germination and growth quantification based on optical density (OD) at the endpoint. Amphotericin B, at the same concentrations used in the anti-yeast assay, was used as the positive control. Plates were incubated at 30 °C for 48 h. Fungal growth was quantified after 48 h by measuring OD at 620 nm using a microplate reader (BioTek Synergy^TM^ MX, Winooski, VT, USA) to determine the OD-based endpoint. Percentage inhibition was calculated relative to untreated controls. Spore suspensions of *A. oryzae* were prepared at the same density for the self-inhibition assay following the anti-*A. niger* assay protocol.

### 2.8. Statistical Analyses

Statistical analyses were performed using Statistical Package for the Social Sciences (SPSS) version 11.5 for Windows (SPSS Inc., Chicago, IL, USA). All the experiments were conducted in three independent biological replicates. The results are presented as the mean ± SD. Data were evaluated using one-way analysis of variance (ANOVA). Pairwise comparisons were performed using Duncan’s multiple range test (DMRT). Differences were considered statistically significant at *p* < 0.05 or *p* < 0.01, as indicated in Results and Discussion.

## 3. Results and Discussion

### 3.1. Establishment of a Heterologous Protein Secretion System Using Endogenous Signal Peptide Sequences

We first examined whether endogenous SPs from *A. oryzae* could stabilize nascent polypeptides and efficiently direct them into the secretory pathway. Secreted hydrolytic and oxidative enzymes from *A. oryzae* BCC7051 were identified based on high-expression profiles reported for the reference strain RIB40 [45]. Five candidates, namely, Aoalp1 (oryzin), AoxynB (xylanase), AofaeB2 (feruloyl esterase B2), Aopep (peptidase S28), and AomreA (isoamyl alcohol oxidase) were selected. Their N-terminal SPs were analyzed using SignalP 6.0 (Table 1). All the predicted SPs ranged from 19 to 24 amino acids in length and exhibited high signal peptide I prediction scores (≥0.94). Meanwhile, the control sequence SPAnglaA (glucoamylase A from *A. niger*) had a substantially lower score of 0.59. Consistent with the canonical SP architecture described in *S. cerevisiae* [46], residues 8 to 12 of the predicted SPs formed a hydrophobic core enriched in leucine, valine, isoleucine, or alanine. At the C-terminus, the conserved −3/−1 AXA or VXA motif associated with signal peptidase I cleavage was evident in all SPs, with alanine frequently occupying the −1 position. SPAoalp1 differed from this by containing glycine at the predicted cleavage site. To functionally assess secretion efficiency, each SP was fused in-frame to the *mgfp* reporter gene. The corresponding plasmid constructs are shown in Figure S1. Correct assembly and reading-frame continuity were confirmed by DNA sequencing. These constructs enabled the evaluation of endogenous SP-driven targeting heterologous proteins to the *A. oryzae* secretory pathway. 

### 3.2. Extracellular GFP Secretion Mediated by Endogenous Signal Peptides

**Figure 2:**
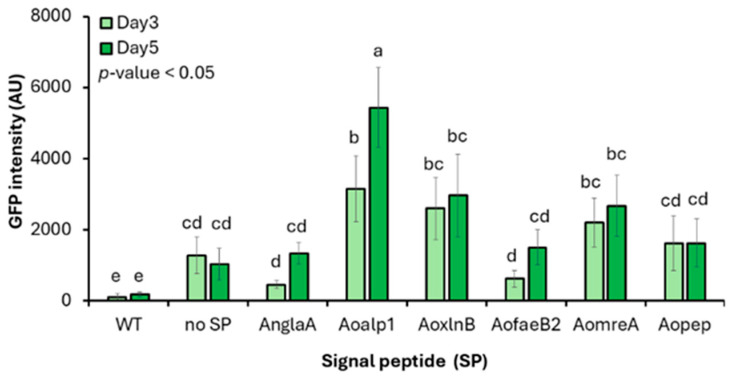
Extracellular secretion of GFP mediated by different signal peptides in *A. oryzae*. GFP fluorescence intensity was measured in culture supernatants of *A. oryzae* transformants expressing *mgfp* fused to the indicated signal peptides after 3 and 5 days of cultivation. Values are expressed as the mean ± standard deviation (*n* = 6 independent transformants). Different lowercase letters indicate statistically distinct groups based on one-way ANOVA followed by DMRT (*p* < 0.05). Abbreviations: GFP, green fluorescent protein; AU, arbitrary units; WT, wild type; SP, signal peptide; no SP, construct lacking a signal peptide; *mgfp*, monomeric green fluorescent protein reporter gene; DMRT, Duncan’s multiple range test.

To assess the ability of endogenous SPs to direct heterologous proteins into the secretory pathway, extracellular GFP secretion was quantified in *A. oryzae* transformants expressing *mgfp* fused to each SP (Figure 2). The no-SP construct forms a negative control representing background/trace release. Fold changes were interpreted as extracellular accumulation enabled by ER targeting via the SP relative to the no-SP control. All the SP-containing constructs produced higher extracellular GFP levels than the SP-free control (Table 2). After 5 d of cultivation, SPAoalp1, SPAoxynB, and SPAomreA increased GFP fluorescence 5.50-, 3.15-, and 2.61-fold, respectively. Meanwhile, the remaining SPs showed more modest effects. SPAoalp1 and SPAoxynB both outperformed SPAnglaA, a heterologous SP derived from *A. niger* that is commonly used for protein secretion [47,48]. This resulted in 4.25- and 2.43-fold higher GFP secretion, respectively. The relatively low signal peptide I score predicted for SPAnglaA may partly account for its weaker performance in *A. oryzae*. Examination of SP sequence features showed that most peptides contained the canonical alanine at the −1 position of the signal peptidase I cleavage motif. In contrast, SPAoalp1 carries a glycine at this position. Although both residues are small and non-polar, glycine confers greater conformational flexibility. This may facilitate access of signal peptidase I to the cleavage site. The superior performance of SPAoalp1 suggests that residue identity at the −1 position can influence SP cleavage efficiency and secretion output.

These data identify SPAoalp1 and SPAoxynB as highly effective endogenous SPs for mediating heterologous protein secretion in *A. oryzae*. Based on its consistently superior performance, SPAoalp1 was selected for the development of the enhanced secretion system.

### 3.3. Enhancing Protein Secretion by Overexpression of Vesicle Trafficking Regulators

Intracellular trafficking and vesicle–membrane fusion are essential for efficient protein secretion in filamentous fungi. These processes depend on the coordinated action of SNARE proteins and SM family regulators. SNARE proteins function as membrane-embedded drivers of vesicle targeting and fusion. Meanwhile, SM proteins transiently associate with target membranes or the cytoplasm to control SNARE complex assembly and activity. These components determine the efficiency of cargo delivery from the ER to the GA and to the extracellular space. Four VTP genes, namely, *bet1*, *sso1*, *sly1*, and *sec1*, were identified in *A. oryzae* BCC7051 based on homology to *S. cerevisiae* and other filamentous fungi [23,49,50,51,52]. Although these proteins shared modest sequence identity with yeast orthologs (27–37%), they were highly conserved among *Aspergillus* species (>75%), suggesting lineage-specific adaptation of vesicle trafficking components (Table 3). Aobet1 (168 amino acids) and Aosso1 (303 amino acids) each contained a single transmembrane domain. This is consistent with their classification as v-SNARE and plasma membrane/septal t-SNARE proteins, respectively. This is in agreement with previous localization studies [50,53]. In contrast, Aosly1 (704 amino acids) and Aosec1 (692 amino acids) proteins lacked transmembrane domains and conformed to the SM protein family. This functions as a chaperone regulating ER–GA trafficking and SNARE complex assembly [23,54,55].

**Figure 3:**
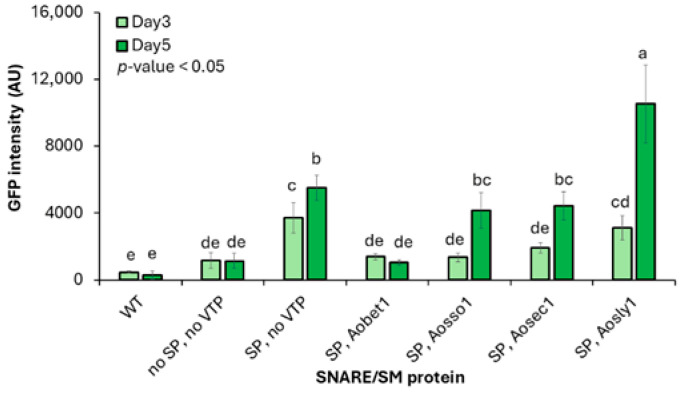
Effects of vesicle trafficking protein overexpression on extracellular GFP secretion in *A. oryzae*. GFP fluorescence intensity was measured in culture supernatants of *A. oryzae* transformants expressing *mgfp* fused to the oryzin signal peptide, with or without co-overexpression of the indicated SNARE or SM family vesicle trafficking proteins, after 3 and 5 d of cultivation. Values are presented as the mean ± standard deviation (*n* = 6 independent transformants). Different lowercase letters indicate statistically distinct groups based on one-way ANOVA followed by DMRT (*p* < 0.05). Abbreviations: GFP, green fluorescent protein; VTP, vesicle trafficking protein; AU, arbitrary units; WT, wild-type; SP, signal peptide; no SP, construct lacking a signal peptide; no VTP, constructs lacking a vesicle trafficking protein; SNARE, soluble N-ethylmaleimide-sensitive factor attachment protein receptor; SM, Sec1/Munc18; *mgfp*, monomeric green fluorescent protein reporter gene; DMRT, Duncan’s multiple range test.

Based on the logic that VTPs act on protein cargos that have already entered the ER, the secretion efficiency was evaluated in the context of the best-performing SP module (SPAoalp1-GFP). Their function was downstream of the ER targeting. To assess whether these VTPs influence secretion efficiency, each gene was co-overexpressed with the SPAoalp1-mgfp construct (Figure S2). The evaluation was quantified as an additional fold increase relative to the SP-only construct. Among the tested regulators, only the overexpression of *Aosly1* in combination with the *SPAoalp1* (*SPAoalp1 + Aosly1*) resulted in a pronounced enhancement of GFP secretion, particularly after 5 d of cultivation. Fluorescence intensity increased approximately 1.97-fold relative to the SP-only construct (SP, no VTP; 10,520.95 ± 2321.11 AU vs. 5338.60 ± 1056.62 AU) as a negative control. It increased approximately 10-fold compared with the system lacking both the SP and VTP (no SP, no VTP; 1022.07 ± 447.06 AU; Figure 3) as a negative control. The upregulation of *Aosly1* gene expression was confirmed by RT-PCR analysis, which showed an increase in transcript levels in the SPAoalp1 + Aosly1 strain (Figure S4).

Among the regulators tested in this study, overexpression of the SM protein Aosly1 resulted in a pronounced enhancement of GFP secretion, consistent with a previous finding of heterologous protein secretion improvement in *S. cerevisiae* [23]. A plausible explanation is that Aosly1 facilitates the transport of modified proteins from the ER to the GA, thereby alleviating ER stress and improving secretion efficiency. In contrast, overexpression of another SM protein, Aosec1, and the selected SNARE proteins did not improve secretion, despite prior evidence of positive effects in yeast and *Trichoderma reesei* [23,53,56,57]. Our results indicate that the effects of individual SNARE and SM components on protein secretion are component-specific, even within the same protein family, and are strongly influenced by host-dependent regulatory mechanisms. We hypothesize that the lack of secretion enhancement by the overexpression of certain SM or SNARE proteins may be associated with changes in the stoichiometry of SNARE/SM complexes, which may affect vesicle trafficking or cargo delivery to productive exocytosis. Taken together, our findings highlight the importance of component-specific and host-dependent regulation of protein secretion and suggest that strategies effective in one host may not be directly generalized across microorganisms.

We identified Aosly1 as the principal vesicle trafficking regulator capable of augmenting protein secretion in *A. oryzae*. Through its role in ER–GA trafficking and SNARE complex assembly and fusion, Aosly1 acts synergistically with the oryzin signal peptide to achieve an approximately 10-fold increase in heterologous protein secretion relative to the native secretory system. Therefore, targeted manipulation of vesicle trafficking regulators is an effective strategy for enhancing secretory capacity in filamentous fungal biofactories.

### 3.4. Validation of the Engineered Secretion System for Heterologous Protein Secretion in A. oryzae

To validate the engineered secretion system, we examined the extracellular production of medium-sized recombinant bovine κ-casein, a food-relevant mammalian complex protein that requires proper eukaryotic processing through the secretory pathway. The engineered system was evaluated using PAFB. This is a small cysteine-rich and well-studied class-B antifungal protein whose antifungal spectrum depends on correct disulfide bond-dependent folding within the secretory pathway during passage. The different structures and sizes of the protein targets allowed us to evaluate the performance of the developed platform for a broad range of secretion and folding requirements. Selecting proteins from phylogenetically distinct donors, that is, bovine and *P. chrysogenum*, rather than *Aspergillus*-derived proteins, challenged the secretion platform and showed that its applicability is broader and not limited to host–donor proximity.

#### 3.4.1. Secretion of Recombinant Aoκ-Casein

The *SPAoalp1–Aocsn3* fusion cassette was co-overexpressed with *Aosly1* in an expression backbone plasmid. Control constructs expressing *SPAoalp1–Aocsn3* or full-length *Aocsn3* alone were also generated (Figure S3A–C). Correct assembly of all plasmids was confirmed by DNA sequencing. Following transformation, RT-PCR verified *Aocsn3* transcription in all transformants (Figure S5). The growth profile of the engineered strain was indistinguishable from that of the wild type, with dry weights of 14–15 g/L after 3 d of cultivation and no detectable residual glucose. Neither SP incorporation nor *Aosly1* overexpression imposed a measurable physiological burden (Figure S6).

**Figure 4:**
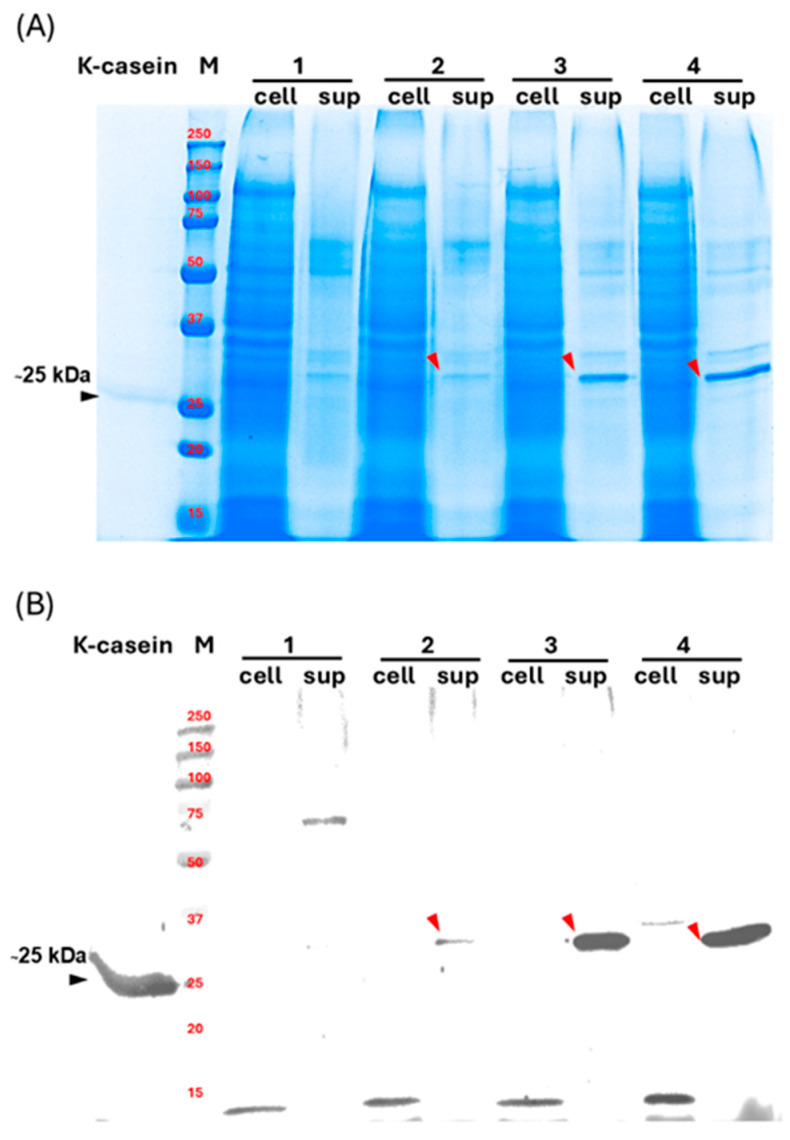
Analysis of Aoκ-casein production in recombinant *A. oryzae*. (**A**) Protein separation by SDS-PAGE. (**B**) Western blot analysis using a bovine κ-casein polyclonal antibody. Lane bovine κ-casein: 1 µg of κ-casein standard (~25 kDa); lane M: prestained protein molecular weight marker; Lanes 1–4 cell: intracellular protein extracts from the wild type, Aoκ-casein, SPAoalp1-Aoκ-casein, and SPAoalp1-Aoκ-casein + Aosly1 strains, respectively; lanes 1–4 sup: corresponding extracellular protein extracts from the same strains. The black arrow indicates the κ-casein standard, and red arrowheads indicate Aoκ-casein produced by recombinant strains. Each lane was loaded with 50 µg of intracellular protein or 20 µg of extracellular protein. Abbreviations: Aoκ-casein, *A. oryzae*–derived κ-casein; SDS-PAGE, sodium dodecyl sulfate–polyacrylamide gel electrophoresis.

SDS-PAGE (Figure 4A) and western blot analysis showed specific κ-casein signals in concentrated culture supernatants (Figure 4B, lanes 2–4 sup). Strains expressing the endogenous *SPAoalp1* (Figure 4B, lane 3 sup) exhibited increased secretion relative to the SP-free construct (Figure 4B, lane 2 sup). Meanwhile, co-overexpression of *Aosly1* further enhanced secretion (Figure 4B, lane 4 sup). Quantitative analysis of Aoκ-casein by ELISA indicated that κ-casein levels increased approximately 1.3-fold relative to the SP-only system (0.24 ± 0.02 mg/L vs. 0.18 ± 0.03 mg/L). They increased approximately 2-fold compared with the strain lacking both *SPAoalp1* and *Aosly1* co-overexpression (0.24 ± 0.02 mg/L vs. 0.11 ± 0.01 mg/L). Endogenous SPAoalp1 enhances secretion efficiency and Aosly1 further augments secretory throughput. Although *Aocsn3* transcripts were detected in the SP-free strain (Figure S5, lane 2), secreted κ-casein was barely detectable (Figure 4B, lane 2 sup). This suggests that there was intracellular degradation or inefficient entry into the secretory pathway. This observation supports the role of endogenous SPs in stabilizing nascent polypeptides and facilitating efficient ER targeting.

Western blot analysis showed that recombinant Aoκ-casein migrated at approximately 30 kDa, exceeding the molecular mass of native bovine κ-casein (Figure 4B, lanes 2–4 and lane κ-casein, respectively). κ-casein undergoes multiple PTMs, including glycosylation and phosphorylation [37]. Differences in modification patterns likely account for the observed mass increase. Intracellular Aoκ-casein (Figure 4B, lane 4 cell) exhibited a higher apparent molecular weight. This is consistent with incomplete signal peptide processing or retention of pre- or pro-segments prior to secretion.

**Figure 5:**
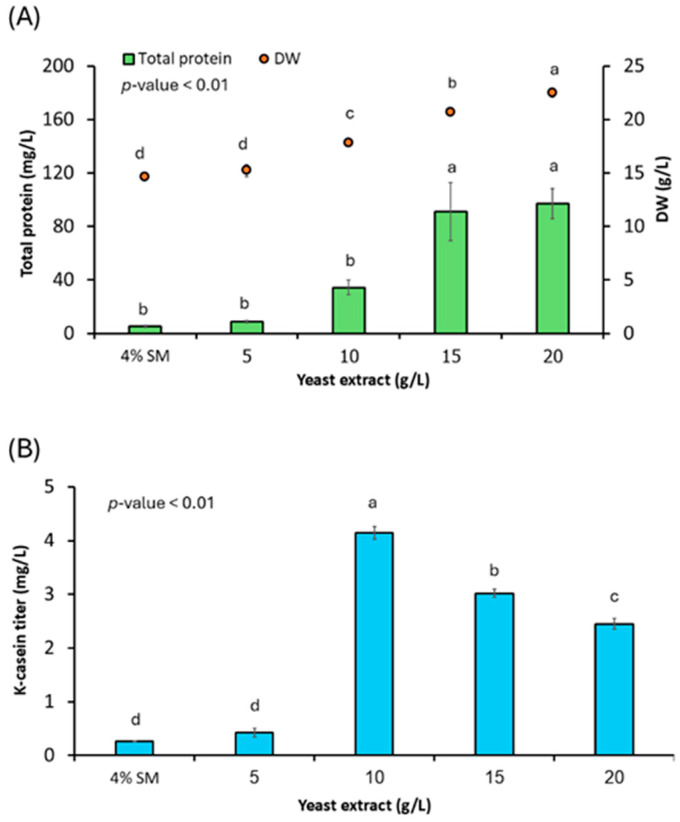
Effect of yeast extract (YE) supplementation on biomass formation and recombinant Aoκ-casein production in *A. oryzae*. (**A**) Biomass accumulation and total extracellular protein concentration in cultures grown with increasing YE concentrations. (**B**) Aoκ-casein titers quantified by ELISA using a bovine κ-casein polyclonal antibody. Values are presented as the mean ± standard deviation (*n* = 3 biological replicates). Bars labeled with different lowercase letters (a–d) indicate statistically distinct groups based on one-way ANOVA followed by DMRT (*p* < 0.01). Abbreviations: DW, dry weight; Aoκ-casein, *A. oryzae*-derived κ-casein; ELISA, enzyme-linked immunosorbent assay; DMRT, Duncan’s multiple range test.

To determine whether secretion can be affected and further enhanced through physiological optimization, nitrogen availability was modulated by supplementing basal semi-synthetic medium with increasing concentrations of yeast extract (YE). Nitrogen sources can affect amino acid supply and protein synthesis and regulate the balance between cell biomass and native and heterologous protein secretion [58,59]. YE is readily assimilated by cells and can be a key nitrogen source for optimal Aoκ-casein secretion. After 3 d of cultivation, both biomass and total protein contents increased with YE concentrations (Figure 5A). However, maximal Aoκ-casein secretion was achieved at 10 g/L YE, reaching 4.14 ± 0.12 mg/L, as quantitated by ELISA (Figure 5B). This represented an approximately 15-fold increase relative to the basal medium (0.27 ± 0.01 mg/L). The Aoκ-casein titers alongside total extracellular proteins derived from 4% SM medium and various concentrations of YE are shown in Table S3. Here, the highest Aoκ-casein titer relative to total protein ratio is achieved with not exceeding 10 g/L YE. Physiological optimization such as YE at the optimal concentration enables targeted protein secretion. Higher YE concentrations promoted biomass accumulation and secretion of native proteins, such as hydrolytic enzymes. However, it did not further enhance recombinant Aoκ-casein production. This indicates that excessive nitrogen availability redirects cellular resources away from heterologous protein export.

The SP selection (SPAoalp1) and Aosly1-meditaed trafficking are defined as genetic improvements providing a secretion framework that improves secretory throughput under a fixed baseline medium. Physiological optimization can also further influence protein secretion. With optimal concentration, the culture medium can strongly improve the targeted protein secretion capacity. This is determined by the maximal ratio of the targeted protein concentration relative to the total protein content. However, exceeding optimal concentration may limit the targeted secretion while allowing secretion of other native proteins. Physiological optimization based on nitrogen sources as an example of YE and its availability could be generalized to apply to other heterologous protein secretion in *A. oryzae.* This is because nitrogen is a basal strong requirement for cell growth and protein production. However, protein-specific optima should be carefully considered for practical application.

#### 3.4.2. Secretion of ~12 kDa Band in Supernatant from AoPAFB-Expressing Strain

**Figure 6:**
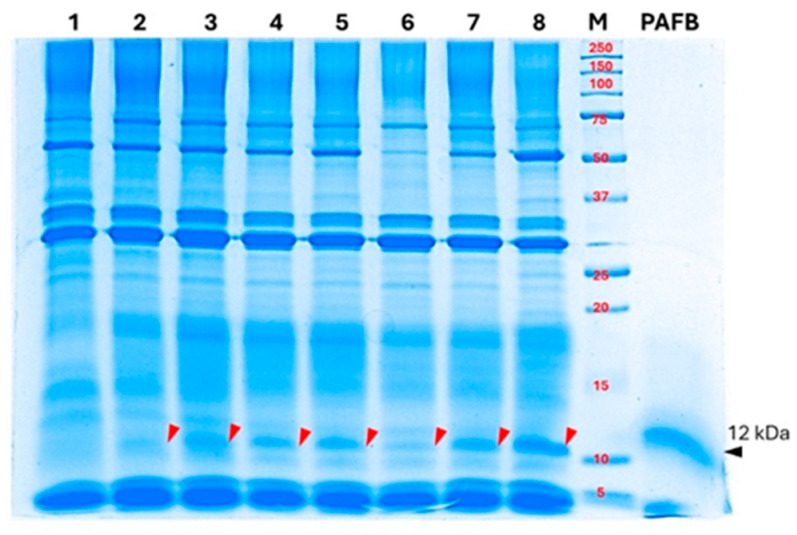
Analysis of a ~12 kDa band detected in the supernatants of AoPAFB-expressing strains. Extracellular proteins were separated by SDS-PAGE using a Tris–glycine system. Lane 1, wild-type *A. oryzae* culture supernatant; lanes 2–8, extracellular protein extracts from independent AoPAFB transformant clones; lane M, prestained protein molecular weight marker; lane PAFB, synthetic PAFB standard (~12 kDa), indicated by a black arrow. The ~12 kDa (putative dimer) detected in recombinant strains is marked by red arrowheads. Each lane was loaded with 50 µg of total protein. Abbreviations: PAFB, *Penicillium chrysogenum* antifungal protein B; SDS-PAGE, sodium dodecyl sulfate–polyacrylamide gel electrophoresis.

A plasmid construct encoding *SPAoalp1–AopafB + Aosly1* was generated (Figure S3D) and introduced into *A. oryzae* to obtain the AoPAFB strain. Transcription of *AopafB* was confirmed using RT–PCR (Figure S7). The secreted protein was analyzed in culture supernatants collected after 3 d of cultivation. SDS-PAGE using a standard Tris-glycine system showed a prominent band at approximately 12 kDa in all AoPAFB transformants (Figure 6, lanes 2–8). This is consistent with the expected size of the PAFB standard (Figure 6, lane PAFB). No corresponding protein band was detected in the wild-type supernatant (Figure 6, lane 1). The bands consistent with a putative PAFB product also migrated at approximately 12 kDa in the Tris-tricine system under strong reducing conditions (Figure S8). The observed molecular weights of the putative PAFB product and the standard were approximately twice the theoretical molecular weight of monomeric PAFB (6.49 kDa). This suggests a stable dimeric or associated form of PAFB and/or anomalous migration, rather than monomer migration, caused by incomplete reduction during sample preparation. This could result in intra- or intermolecular disulfide bond formation [60]. Therefore, the apparent molecular weight of PAFB on SDS-PAGE may deviate from the theoretical mass. However, this study has some limitations. The definitive molecular identity at the sequence level was not determined and the observed band is referred to as a ~12 kDa extracellular band consistent with *AopafB* gene transcription from the AoPAFB-expressing strain, based on its electrophoresis appearance and transcriptional evidence. The definitive protein identification with amino acid sequence confirmation, including matrix-assisted laser desorption/ionization time-of-flight mass spectrometry (MALDI-TOF MS), electrospray ionization mass spectrometry (ESI-MS), Edman degradation, immunodetection by western blot analysis or ELISA based on the PAFB-specific antibody, should be a focus in future studies.

The antifungal activity in the culture supernatants of the AoPAFB-expressing strain was evaluated using broth microdilution. Modified semi-synthetic medium optimized for Aoκ-casein production was used. Twenty-five times concentrated culture supernatant from the recombinant strain grown for 3 d was serially diluted to final test concentrations ranging from 1.56 to 50% (*v*/*v*), and MIC_90_ values were determined. Supernatant, in which a ~12 kDa band was detected, exhibited inhibitory activity against both yeast and filamentous fungal pathogens (Table 4). There were MIC_90_ values of 4.56 ± 0.17% and 8.24 ± 1.81% (*v*/*v*) against two *C. albicans* isolates and 4.68 ± 2.04% (*v*/*v*) against *A. niger*. In contrast, no inhibitory activity was observed for the wild-type culture supernatant. The PAFB standard also showed stronger antifungal activity against *A. niger* (MIC_90_ = 1.38 ± 0.03 µg/mL) than the *C. albicans* strains (3.61 ± 1.58 and 5.95 ± 1.02 µg/mL) in all the pathogen tests, in accordance with its reported antifungal spectrum. A limitation of this study is the absence of control strains, for example, an empty-vector control or an irrelevant-protein expression strain. This prevents definitive attribution of the observed activity to the recombinant protein. Future work will include control strains to distinguish cassette-specific effects from general transformation effects, and protein purification with activity assessment to confirm the protein-specific effect.

Analysis of the culture supernatant from the AoPAFB-expressing strain reveals a ~12 kDa band, consistent with the gene expression evidence, and co-migrates with the concurrently run standard, suggesting potential protein secretion by the engineered strain. However, the antifungal activity observed in the supernatant of the AoPAFB-expressing strain cannot be attributed solely to AoPAFB, as heterologous gene expression may exert secondary effects on host cellular physiology, thereby altering the secretome as well as other metabolites and proteins. Consequently, the contribution of AoPAFB to the observed bioactivities cannot be definitively determined from the crude supernatant alone. Moreover, the definitive molecular identity of the protein has not yet been confirmed. These findings therefore provide only preliminary evidence, and further purification together with MS and Western blot–based identification would be required to establish direct causality.

Assessment of host susceptibility to address potential autotoxicity or stress-response effects was performed by a self-inhibition assay using *A. oryzae* BCC7051 as the target. No inhibitory effect on the fungal host was observed for the supernatant from the AoPAFB-expressing strain, nor for the PAFB standard or wild-type supernatant (Table 4). These results indicate no autotoxicity of the supernatant from the AoPAFB-expressing strain under the assay conditions. Apart from general self-defense mechanisms, for example, cell wall/membrane composition and transport pathways, the fungal host insensitivity might be explained by the calcium-mediated resistance mechanism. The mechanism has been identified in *Aspergillus flavus* against PgAFP, a protein identical to the mature PAFB [61]. Although the putative *pafB* gene was not identified in *A. oryzae* BCC7051 and the fungus was not a native producer of PAFB, its close genetic evolution relationship to the resistant strain like *A. flavus* suggests it may possess inherent calcium-mediated resistance mechanisms to this class of compounds. A Detailed study of the resistance mechanism might be further explored in *A. oryzae.*

**Figure 7:**
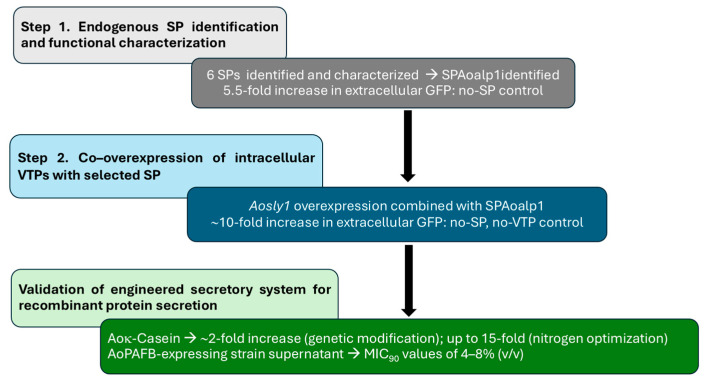
Summary of the workflow schematic of the two-step engineered targets for improving carrier-free protein secretion with integrative outcomes. Step 1 is quantified as a fold increase relative to the no-SP control and Step 2 as the additional fold increase relative to the SP-only construct. Validation of the engineered secretory system was complemented through extracellular bovine Aoκ-casein and supernatant from AoPAFB-expressing strain. Abbreviations: GFP, green fluorescent protein; SP, signal peptide; VTPs, vesicle trafficking proteins; Aoκ-casein, *A. oryzae*-derived κ-casein; PAFB, *Penicillium chrysogenum* antifungal protein B; MIC_90_, Minimum inhibitory concentration required to inhibit 90% of growth.

Taken together, our platform provides a valuable basis for future studies into peptide or protein folding, post-translational modifications, and secretion in filamentous fungi, and for comparative analyses of functional protein variants produced using engineered fungal biofactories. Our results demonstrate that two engineering steps of protein secretion enable extracellular protein production of Aoκ-casein and provide initial evidence that the platform supports secretion of a putative small cysteine-rich antifungal protein. The platform facilitates extracellular protein production in the industrial strain of *A. oryzae* without requiring a carrier fusion partner. A summary of the results is shown in Figure 7.

## 4. Conclusions

The secretory capacity of *A. oryzae* can be strongly enhanced through targeted genetic engineering of both signal peptide recognition and intracellular trafficking. Among the endogenous signal peptides evaluated, oryzin-derived SPAoalp1 markedly improved the secretion of the GFP reporter. This increased extracellular levels 5.5-fold relative to constructs lacking an SP. Co-overexpression of *Aosly1*, an SM family regulator of SNARE-mediated trafficking, further elevated GFP secretion by approximately two-fold relative to the SPAoalp1-control and approximately 10-fold relative to that of the no-SP control. This established a synergistic effect between SP-mediated ER targeting and optimized vesicle fusion. Application of the engineered system to heterologous eukaryotic proteins demonstrated its feasibility. Based on genetic improvements, secretion of recombinant Aoκ-casein increased approximately 2-fold relative to the strain lacking both SP and *Aosly1* co-overexpression and reached a 15-fold increase following physiological optimization. The engineered system further enabled secretion of a ~12 kDa extracellular band consistent with *AopafB* gene transcription from the AoPAFB-expressing strain. This yielded culture supernatant with inhibitory activity against yeast and filamentous fungal pathogens. These findings show previously unrecognized contributions of endogenous SP architecture and SM-protein–mediated trafficking to secretion efficiency in *A. oryzae*. This represents a mechanistic advance beyond prior approaches that relied on carrier fusion proteins or disruption of secretory pathways.

Combining endogenous SP selection with targeted manipulation of a vesicle trafficking regulator provides an effective carrier-free strategy to enhance heterologous protein secretion in *A. oryzae*. While our data is consistent with improved ER targeting and secretory throughput, detailed mechanistic determinants, for example, effects on folding quality control or specific trafficking steps, remain to be established. The system offers a modular framework for engineering fungal secretion and expands the utility of *A. oryzae* as an industrial platform for producing diverse recombinant proteins; when bioactivity is evaluated, purification and molecular confirmation will strengthen causal interpretation.

## Figures and Tables

**Table 1:** Predicted signal peptide sequences and features from *A. oryzae* BCC7051. Putative signal peptides (SPs) from *A. oryzae* BCC7051 were identified using SignalP 6.0 to predict N-terminal signal sequences, signal peptidase I cleavage sites, and Sec/SPI secretion probability scores.

Signal Peptide (SP)	Accession ID	Amino Acid Sequence (N-Terminus)	Length (aa)	Sec/SPI Prediction Score	Cleavage Site	Cleavage Probability
SPAnglaA (Glucoamylase A)	CAA25219.1	MSFRSLLALSGLVCTGLA	18	0.5934	A–N	0.3139
SPAoalp1 (Oryzin/alkaline protease)	OOO09972.1	MQSIKRTLLLLGAILPAVLG	20	0.9996	G–A	0.9709
SPAoxynB (Xylanase G1)	OOO09458.1	MVSFSSLLLAVSAVSGALA	19	0.9421	A–A	0.8294
SPAofaeB2 (Feruloyl esterase B2)	OOO12167.1	MKVSLWLTLLGVNLSLALA	19	0.9904	A–V	0.6090
SPAomreA (Isoamyl alcohol oxidase)	OOO13350.1	MPSLSTLKLGAFLGLAAIAPLIEA	24	0.9961	A–A	0.9088
SPAopep (Peptidase S28)	OOO13707.1	MQFLPPLSIVTLLASWPSLSRA	22	0.9545	A–I	0.5701

Abbreviations: SP, signal peptide; Sec/SPI, signal peptide I; A, alanine; N, asparagine; G, glycine; V, valine; I, isoleucine; L, leucine.

**Table 2:** Relative fluorescence intensity of secreted GFP in *A. oryzae* transformants expressing different signal peptides after 5 d of cultivation.

Signal Peptide (SP)	Fold Increase vs. No SP	Fold Increase vs. SPAnglaA
No SP	1.00	0.77
SPAnglaA (Glucoamylase A)	1.29	1.00
SPAoalp1 (Oryzin)	5.50	4.25
SPAoxynB (Xylanase G1)	3.15	2.43
SPAofaeB2 (Feruloyl esterase B2)	1.47	1.13
SPAomreA (Isoamyl alcohol oxidase)	2.61	2.01
SPAopep (Peptidase S28)	1.58	1.22

The relative fluorescence intensity of secreted GFP was measured in culture supernatants of *A. oryzae* transformants expressing different signal peptides (SPs). Values are expressed as fold increases relative to either the no-SP construct (negative control) or the benchmark SP from *A. niger* (SPAnglaA). Abbreviations: GFP, green fluorescent protein; SP, signal peptide.

**Table 3:** Vesicle trafficking proteins identified in *A. oryzae* BCC7051.

Protein Family	Accession ID	Protein Name	Length (aa)	Transmembrane Domains (Residues)	Predicted Subcellular Localization	Amino Acid Identity Among *Aspergillus* spp. (%)
SNARE	OOO11641.1	Aobet1	168	1 (150–167)	GA membrane	78.74–99.40
	OOO07523.1	Aosso1	303	1 (281–298)	Plasma membrane, septum	78.45–99.67
SM	OOO06293.1	Aosly1	704	0	Between ER to Golgi trafficking	84.56–99.86
	OOO07634.1	Aosec1	692	0	Between Golgi to plasma membrane trafficking	76.96–99.56

SNARE and Sec1/Munc18 (SM) family vesicle trafficking proteins were identified in *A. oryzae* BCC7051 based on sequence homology to characterized fungal trafficking components. Protein sequences were analyzed to determine the transmembrane domain content, predicted localization within the secretory pathway, and amino acid identity relative to orthologs from other *Aspergillus* species. Abbreviations: GA, Golgi apparatus; ER, endoplasmic reticulum; SNARE, soluble N-ethylmaleimide-sensitive factor attachment protein receptor; SM, Sec1/Munc18.

**Table 4:** Antifungal activity of the PAFB standard, AoPAFB-expressing strain supernatant, and wild-type supernatant of *A. oryzae*.

Sample	*C. albicans* ATCC 90028	*C. albicans* ATCC 10231	*A. niger* DMST 15538	*A. oryzae* BCC7051
Amphotericin B (μg/mL)	0.63 ± 0.06	1.83 ± 0.89	1.39 ± 0.55	4.67 ± 0.21
PAFB standard (μg/mL)	3.61 ± 1.58	5.95 ± 1.02	1.38 ± 0.03	No inhibition
AoPAFB-expressing strain supernatant (% *v*/*v*)	4.56 ± 0.17	8.24 ± 1.81	4.68 ± 2.04	No inhibition
Wild-type supernatant (% *v*/*v*)	No inhibition	No inhibition	No inhibition	No inhibition

Antifungal activity was evaluated using the synthetic PAFB protein and culture supernatants from recombinant AoPAFB and wild-type *A. oryzae* strains. MIC_90_ values are expressed as protein concentration (µg/mL) for the PAFB standard or as the lowest tested volume percentage (% *v*/*v*) of 25× concentrated supernatant from the AoPAFB-expressing strain, achieving ≥90% growth inhibition. The MIC_90_ value is defined as the endpoint for antifungal activity in crude supernatants, corresponding to ≥90% inhibition as determined throughout the study. Values represent the mean ± SD of three independent biological replicates. No inhibition was observed for the wild-type supernatant at the highest tested concentration (50% *v*/*v*). Abbreviations: MIC_90_, minimum inhibitory concentration required to inhibit 90% of growth; SD, standard deviation.

## Data Availability

The original contributions presented in the study are included in the article/Supplementary Material. Further inquiries can be directed to the corresponding authors.

## Supplementary Materials

The following supporting information can be downloaded at: https://www.mdpi.com/article/10.3390/jof12040289/s1. Table S1. Overlapping primer sets used for PCR amplification and plasmid construction; Table S2. Oligonucleotide primers used for gene expression analysis; Table S3. Aoκ-casein titers quantified by ELISA, total extracellular protein and Aoκ-casein: total protein ratios from the recombinant strain grown in 4% SM medium or with increasing concentrations of yeast extract (YE); Figure S1. Plasmid map of pSPAoxxx-mgfp used for functional analysis of signal peptides; Figure S2. Plasmid map of pSPAoalp1-mgfp + Aovtp used for functional analysis of vesicle trafficking proteins; Figure S3. Plasmid maps used for heterologous protein expression in *Aspergillus oryzae*; Figure S4. Reverse transcription polymerase chain reaction analysis of *Aosly1* expression in recombinant *Aspergillus oryzae* strains; Figure S5. Reverse transcription polymerase chain reaction analysis of *Aocsn3* gene expression in recombinant *Aspergillus oryzae* strains; Figure S6. Growth characteristics of the recombinant Aoκ-casein strain; Figure S7. Reverse transcription polymerase chain reaction analysis of *AopafB* gene expression in recombinant *Aspergillus oryzae* strains; Figure S8. Analysis of a ~12 kDa band detected in the supernatant from the AoPAFB-expressing strain using a Tris-tricine-SDS buffer system with strongly reducing conditions.

## Abbreviations

The following abbreviations are used in this manuscript:

*A. niger*	*Aspergillus niger*
*A. oryzae*	*Aspergillus oryzae*
Aoκ-casein	*Aspergillus oryzae*–derived κ-casein
AU	Arbitrary units
CD	Czapek Dox medium
DW	Dry weight
ELISA	Enzyme-linked immunosorbent assay
ER	Endoplasmic reticulum
GA	Golgi apparatus
GFP	Green fluorescent protein
GRAS	Generally Recognized as Safe
HRP	Horseradish peroxidase
MIC_90_	Minimum inhibitory concentration required to inhibit 90% of growth
OD	Optical density
PAFB	*Penicillium chrysogenum* antifungal protein B
PBS	Phosphate-buffered saline
PCR	Polymerase chain reaction
PTM	Post-translational modification
RT–PCR	Reverse transcription polymerase chain reaction
SD	Standard deviation
SDS-PAGE	Sodium dodecyl sulfate–polyacrylamide gel electrophoresis
SM	Sec1/Munc18
SNARE	Soluble N-ethylmaleimide-sensitive factor attachment protein receptor
SP	Signal peptide
VTP	Vesicle trafficking protein
YE	Yeast extract

## References

[1] Nguyen T., Karl M., Santini A. (2016). Red Yeast Rice. Foods.

[2] Poirier M., Hugot C., Spatz M., Da Costa G., Lapiere A., Michaudel C., Danne C., Martin V., Langella P., Michel M.-L. (2022). Effects of five filamentous fungi used in food processes on in vitro and in vivo gut inflammation. J. Fungi.

[3] Wang C., Hu C., Li X., Shen R., Yin L., Wu Q., Hu T. (2025). Effects of *Rhizopus oligosporus*-mediated solid-state fermentation on the protein profile and α-glucosidase inhibitory activity of selenium-biofortified soybean tempeh. Foods.

[4] Tong S., Qiu Q., Gao J., Yu J., Xu Y., Liao Z. (2026). Edible fungus *Fusarium venenatum*: Advances, challenges, and engineering strategies for future food production. Metab. Eng..

[5] Singh N., Gaur S., Dai X., Sharma M., Chen J. (2021). GRAS Fungi: A new horizon in safer food product. Fungi in Sustainable Food Production; Fungal Biology.

[6] Barbesgaard P., Heldt-Hansen H.P., Diderichsen B. (1992). On the safety of *Aspergillus oryzae*: A review. Appl. Microbiol. Biotechnol..

[7] Blumenthal C.Z. (2004). Production of toxic metabolites in *Aspergillus niger*, *Aspergillus oryzae*, and *Trichoderma reesei*: Justification of mycotoxin testing in food grade enzyme preparations derived from the three fungi. Regul. Toxicol. Pharmacol..

[8] Lin C.-H., Wei Y.-T., Chou C.-C. (2006). Enhanced antioxidative activity of soybean koji prepared with various filamentous fungi. Food Microbiol..

[9] Cairns T.C., Barthel L., Meyer V. (2021). Something old, something new: Challenges and developments in *Aspergillus niger* biotechnology. Essays Biochem..

[10] Thammarongtham C., Nookaew I., Vorapreeda T., Srisuk T., Land M.L., Jeennor S., Laoteng K. (2018). Genome characterization of oleaginous *Aspergillus oryzae* BCC7051: A potential fungal-based platform for lipid production. Curr. Microbiol..

[11] Tominaga M., Lee Y.H., Hayashi R., Suzuki Y., Yamada O., Sakamoto K., Gotoh K., Akita O. (2006). Molecular analysis of an inactive aflatoxin biosynthesis gene cluster in *Aspergillus oryzae* RIB strains. Appl. Environ. Microbiol..

[12] Sun Z., Wu Y., Long S., Feng S., Jia X., Hu Y., Ma M., Liu J., Zeng B. (2024). *Aspergillus oryzae* as a Cell Factory: Research and Applications in Industrial Production. J. Fungi.

[13] Liu D., Garrigues S., de Vries R.P. (2023). Heterologous protein production in filamentous fungi. Appl. Microbiol. Biotechnol..

[14] Liu L., Feizi A., Österlund T., Hjort C., Nielsen J. (2014). Genome-scale analysis of the high-efficient protein secretion system of *Aspergillus oryzae*. BMC Syst. Biol..

[15] Chutrakul C., Panchanawaporn S., Jeennor S., Anantayanon J., Vorapreeda T., Vichai V., Laoteng K. (2019). Functional characterization of novel U6 RNA polymerase III promoters: Their implication for CRISPR-Cas9-Mediated gene editing in *Aspergillus oryzae*. Curr. Microbiol..

[16] Chutrakul C., Panchanawaporn S., Jeennor S., Anantayanon J., Laoteng K. (2022). Promoter exchange of the cryptic nonribosomal peptide synthetase gene for oligopeptide production in *Aspergillus oryzae*. J. Microbiol..

[17] Jeennor S., Anantayanon J., Chutrakul C., Panchanawaporn S., Laoteng K. (2022). Novel pentose-regulated promoter of *Aspergillus oryzae* with application in controlling heterologous gene expression. Biotechnol. Rep..

[18] Laoteng K., Anantayanon J., Chutrakul C., Panchanawaporn S., Jeennor S. (2023). Transcriptome-based mining of the constitutive promoters for tuning gene expression in *Aspergillus oryzae*. J. Microbiol..

[19] Wang Q., Zhong C., Xiao H. (2020). Genetic engineering of filamentous fungi for efficient protein expression and secretion. Front. Bioeng. Biotechnol..

[20] Mitra N., Sinha S., Ramya T.N.C., Surolia A. (2006). N-linked oligosaccharides as outfitters for glycoprotein folding, form and function. Trends Biochem. Sci..

[21] Adnan M., Islam W., Waheed A., Hussain Q., Shen L., Wang J., Liu G. (2023). SNARE protein Snc1 is essential for vesicle trafficking, membrane fusion and protein secretion in fungi. Cells.

[22] Cairns T.C., Zheng X., Zheng P., Sun J., Meyer V. (2021). Turning inside out: Filamentous fungal secretion and its applications in biotechnology, agriculture, and the clinic. J. Fungi.

[23] Hou J., Tyo K., Liu Z., Petranovic D., Nielsen J. (2012). Engineering of vesicle trafficking improves heterologous protein secretion in *Saccharomyces cerevisiae*. Metab. Eng..

[24] Ohno A., Maruyama J.I., Nemoto T., Arioka M., Kitamoto K. (2011). A carrier fusion significantly induces unfolded protein response in heterologous protein production by *Aspergillus oryzae*. Appl. Microbiol. Biotechnol..

[25] Hoang H.-D., Maruyama J.I., Kitamoto K. (2015). Modulating endoplasmic reticulum-Golgi cargo receptors for improving secretion of carrier-fused heterologous proteins in the filamentous fungus *Aspergillus oryzae*. Appl. Environ. Microbiol..

[26] Nakajima K.I., Asakura T., Maruyama J.I., Morita Y., Oike H., Shimizu-Ibuka A., Misaka T., Sorimachi H., Arai S., Kitamoto K. (2006). Extracellular production of neoculin, a sweet-tasting heterodimeric protein with taste-modifying activity, by *Aspergillus oryzae*. Appl. Environ. Microbiol..

[27] Panchanawaporn S., Chutrakul C., Jeennor S., Anantayanon J., Laoteng K. (2025). Development of *Aspergillus oryzae* BCC7051 as a robust cell factory towards the transcriptional regulation of protease-encoding genes for industrial applications. J. Fungi.

[28] Farrell H.M., Jimenez-Flores R., Bleck G.T., Brown E.M., Butler J.E., Creamer L.K., Hicks C.L., Hollar C.M., Ng-Kwai-Hang K.F., Swaisgood H.E. (2004). Nomenclature of the proteins of cows’ milk--sixth revision. J. Dairy. Sci..

[29] Fox P.F., McSweeney P.L.H. (2013). Advanced Dairy Chemistry: Volume 1A—Proteins: Basic Aspects.

[30] Dalgleish D.G. (2011). On the structural models of bovine casein micelles—Review and possible improvements. Soft Matter..

[31] Garrigues S., Gandía M., Marcos J.F. (2016). Occurrence and function of fungal antifungal proteins: A case study of the citrus postharvest pathogen *Penicillium digitatum*. Appl. Microbiol. Biotechnol..

[32] Holzknecht J., Marx F. (2024). Navigating the fungal battlefield: Cysteine-rich antifungal proteins and peptides from Eurotiales. Front. Fungal Biol..

[33] Huber A., Hajdu D., Bratschun-Khan D., Gáspári Z., Varbanov M., Philippot S., Fizil Á., Czajlik A., Kele Z., Sonderegger C. (2018). New antimicrobial potential and structural properties of PAFB: A cationic, cysteine-rich protein from *Penicillium chrysogenum* Q176. Sci. Rep..

[34] Huber A., Lerchster H., Marx F. (2019). Nutrient excess triggers the expression of the *Penicillium chrysogenum* antifungal protein PAFB. Microorganisms.

[35] Huber A., Galgóczy L., Váradi G., Holzknecht J., Kakar A., Malanovic N., Leber R., Koch J., Keller M.A., Batta G. (2020). Two small, cysteine-rich and cationic antifungal proteins from *Penicillium chrysogenum*: A comparative study of PAF and PAFB. Biochim. Biophys. Acta Biomembr..

[36] Marx F. (2004). Small, basic antifungal proteins secreted from filamentous ascomycetes: A comparative study regarding expression, structure, function and potential application. Appl. Microbiol. Biotechnol..

[37] Hettinga K., Bijl E. (2022). Can recombinant milk proteins replace those produced by animals?. Curr. Opin. Biotechnol..

[38] Pahirulzaman K.A.K., Williams K., Lazarus C.M. (2012). A toolkit for heterologous expression of metabolic pathways in *Aspergillus oryzae*. Methods Enzymol..

[39] Teufel F., Almagro Armenteros J.J., Johansen A.R., Gíslason M.H., Pihl S.I., Tsirigos K.D., Winther O., Brunak S., von Heijne G., Nielsen H. (2022). SignalP 6.0 predicts all five types of signal peptides using protein language models. Nat. Biotechnol..

[40] Hallgren J., Tsirigos K.D., Pedersen M.D., Almagro Armenteros J.J., Marcatili P., Nielsen H., Krogh A., Winther O. (2022). DeepTMHMM predicts alpha and beta transmembrane proteins using deep neural networks. bioRxiv.

[41] Punya J., Tachaleat A., Wattanachaisaereekul S., Haritakun R., Boonlarppradab C., Cheevadhanarak S. (2013). Functional expression of a foreign gene in *Aspergillus oryzae* producing new pyrone compounds. Fungal Genet. Biol..

[42] Chutrakul C., Jeennor S., Panchanawaporn S., Cheawchanlertfa P., Suttiwattanakul S., Veerana M., Laoteng K. (2016). Metabolic engineering of long chain-polyunsaturated fatty acid biosynthetic pathway in oleaginous fungus for dihomo-gamma linolenic acid production. J. Biotechnol..

[43] National Committee for Clinical Laboratory Standards (2018). Methods for Dilution Antimicrobial Susceptibility Tests for Bacteria That Grow Aerobically: Approved Standard.

[44] National Committee for Clinical Laboratory Standards (2002). Reference Method for Broth Dilution Antifungal Susceptibility Testing of Yeasts, Approved Standard-Second Edition.

[45] Oda K., Kakizono D., Yamada O., Iefuji H., Akita O., Iwashita K. (2006). Proteomic analysis of extracellular proteins from *Aspergillus oryzae* grown under submerged and solid-state culture conditions. Appl. Environ. Microbiol..

[46] Xue S., Liu X., Pan Y., Xiao C., Feng Y., Zheng L., Zhao M., Huang M. (2023). Comprehensive analysis of signal peptides in *Saccharomyces cerevisiae* reveals features for efficient secretion. Adv. Sci..

[47] Fleissner A., Dersch P. (2010). Expression and export: Recombinant protein production systems for *Aspergillus*. Appl. Microbiol. Biotechnol..

[48] Ogino C., Matsuda T., Okazaki F., Tanaka T., Kondo A. (2014). The effect of combining signal sequences with the N28 fragment on GFP production in *Aspergillus oryzae*. Process Biochem..

[49] Stone S., Sacher M., Mao Y., Carr C., Lyons P., Quinn A.M., Ferro-Novick S. (1997). Bet1p activates the v-SNARE Bos1p. Mol. Biol. Cell.

[50] Kuratsu M., Taura A., Shoji J.Y., Kikuchi S., Arioka M., Kitamoto K. (2007). Systematic analysis of SNARE localization in the filamentous fungus Aspergillus oryzae. Fungal Genet. Biol..

[51] Valkonen M., Kalkman E.R., Saloheimo M., Penttilä M., Read N.D., Duncan R.R. (2007). Spatially segregated SNARE protein interactions in living fungal cells. J. Biol. Chem..

[52] Kwon M.J., Arentshorst M., Fiedler M., de Groen F.L.M., Punt P.J., Meyer V., Ram A.F.J. (2014). Molecular genetic analysis of vesicular transport in *Aspergillus niger* reveals partial conservation of the molecular mechanism of exocytosis in fungi. Microbiology.

[53] Van Zyl J.H.D., den Haan R., Van Zyl W.H. (2016). Overexpression of native *Saccharomyces cerevisiae* ER-to-Golgi SNARE genes increased heterologous cellulase secretion. Appl. Microbiol. Biotechnol..

[54] Kosodo Y., Noda Y., Adachi H., Yoda K. (2002). Binding of Sly1 to Sed5 enhances formation of the yeast early Golgi SNARE complex. J. Cell Sci..

[55] Duan M., Gao G., Lin A., Mackey E.J., Banfield D.K., Merz A.J. (2024). SM protein Sly1 and a SNARE Habc domain promote membrane fusion through multiple mechanisms. J. Cell Biol..

[56] Wu Y., Sun X., Xue X., Luo H., Yao B., Xie X., Su X. (2017). Overexpressing key component genes of the secretion pathway for enhanced secretion of an *Aspergillus niger* glucose oxidase in *Trichoderma reesei*. Enzyme Microb. Technol..

[57] Ji W., Wang X., Liu X., Wang Y., Liu F., Xu B., Luo H., Tu T., Zhang W., Xu X. (2023). Combining manipulation of integration loci and secretory pathway on expression of an *Aspergillus niger* glucose oxidase gene in *Trichoderma reesei*. Microb. Cell Fact..

[58] Tudzynski B. (2014). Nitrogen regulation of fungal secondary metabolism in fungi. Front. Microbiol..

[59] Yang Y., Li Y., Zhu J. (2024). Research progress on the function and regulatory pathways of amino acid permeases in fungi. World J. Microbiol. Biotechnol..

[60] Victorio C.G., Sawyer N. (2023). Folding-assisted peptide disulfide formation and dimerization. ACS Chem. Biol..

[61] Delgado J., Owens R.A., Doyle S., Núñez F., Asensio M.A. (2017). Quantitative proteomics reveals new insights into calcium-mediated resistance mechanisms in *Aspergillus flavus* against the antifungal protein PgAFP in cheese. Food Microbiol..

