# Supplementary material for: Two-Step Engineering of Food-Grade Aspergillus oryzae via Endogenous Signal Peptides and Vesicle Trafficking Proteins to Enhance Carrier-Free Protein Secretion
Source: J Fungi (Basel). 2026 Apr 18;12(4):289. doi: 10.3390/jof12040289 (PMC13117078; doi:10.3390/jof12040289)
Supplement: Supplementary file 1 [file jof-12-00289-s001.zip › jof-4214711-supplementary.pdf]

Two-Step Engineering of Food-Grade *Aspergillus oryzae* via Endogenous Signal Peptides and Vesicle Trafficking Proteins to Enhance Carrier-Free Protein Secretion

Sarocha Panchanawaporn [1], Nakul Rattanaphan [1], Sukanya Jeennor [1], Jutamas Anantayanon [1], Weerapong Woraprayote [2,†], Laphaslada Pumpuang [2,‡], Thipphiya Karirat [1,§], Nuttamon Prompakdee [1], Kobkul Laoteng [1] and Chanikul Chutrakul [1,*]

[1] Industrial Bioprocess Technology Research Team (IIBT), Functional Ingredients and Food Innovation Research Group (IFIG), National Center for Genetic Engineering and Biotechnology (BIOTEC), National Science and Technology Development Agency (NSTDA), Thailand Science Park, Phahonyothin Road, Khlong Nueng, Khlong Luang, Pathum Thani 12120, Thailand; sarocha.pan@biotec.or.th (S.P.); nakul.rat@biotec.or.th (N.R.); sukanya.jee@biotec.or.th (S.J.); jutamas.ana@biotec.or.th (J.A.); thipphiya020@gmail.com (T.K.); nut57nuttamon@gmail.com (N.P.); kobkul@biotec.or.th (K.L.)

[2] Food Biotechnology Research Team, Functional Ingredients and Food Innovation Research Group (IFIG), National Center for Genetic Engineering and Biotechnology (BIOTEC), National Science and Technology Development Agency (NSTDA), Thailand Science Park, Phahonyothin Road, Khlong Nueng, Khlong Luang, Pathum Thani 12120, Thailand; weerapong.wor@mahidol.ac.th (W.W.); laphaslada.pum@biotec.or.th (L.P.)

[*] Correspondence: chanikul@biotec.or.th; Tel.: +66-2-5646700 (ext. 3769); Fax: +66-2-5646707

[†] Present address: Department of Biochemistry, Siriraj Metabolomics and Phenomics Center (SiMPC), Faculty of Medicine Siriraj Hospital, Mahidol University, Bangkok 10700, Thailand.

[‡] Present address: Applied Peptides and Proteomics Research Team, Functional Ingredients and Food Innovation Research Group (IFIG), National Center for Genetic Engineering and Biotechnology (BIOTEC), National Science and Technology Development Agency (NSTDA), Thailand Science Park, Phahonyothin Road, Khlong Nueng, Khlong Luang, Pathum Thani 12120, Thailand.

[§] Present address: Thaiva Laboratories Co., Ltd. 40/4 Samkhok-Sena Road, Taiy Koh, Samkhok, Pathum Thani 12160, Thailand.

Table S1. Overlapping primer sets used for PCR amplification and plasmid construction

Plasmid	Amplified fragment	Forward primer	Sequence (5′–3′)	Reverse primer	Sequence (5′–3′)
pmgfp	*mgfp*	mgfp_F	ATGAGTAAAGGAGAAGAACTT	mgfp_R	CTTCTTAATTGTGTCAGCAACTTAATGATGGTGATGATGGTG
pSPAnglaA-mgfp	*SPAnglaA*	SPAnglaA_F	taccgaagaacgcaGCGGCCGCATGTCGTTCCGATCTCTACT	SSAnglaA_R	AAGTTCTTCTCCTTTACTCATTGCCAACCCTGTGCAGACGAG
	mgfp	mgfp_F	ATGAGTAAAGGAGAAGAACTT	mgfp_R	CTTCTTAATTGTGTCAGCAACTTAATGATGGTGATGATGGTG
pSPAoalp1-mgfp	*SPAoalp1*	SPAoalp1_F	taccgaagaacgcaGCGGCCGCATGCAGTCCATCAAGCGTAC	SSAoalp1_R	AAGTTCTTCTCCTTTACTCATGGCACCGAGGACCGCGGGA
	mgfp	mgfp_F	ATGAGTAAAGGAGAAGAACTT	mgfp_R	CTTCTTAATTGTGTCAGCAACTTAATGATGGTGATGATGGTG
pSPAoxynB-mgfp	*SPAoxynB*	SPAoxynB_F	taccgaagaacgcaGCGGCCGCATGGTTAGCTTCTCTTCTCTC	SSAoxlnB_R	AAGTTCTTCTCCTTTACTCATAGCCAAAGCGCCGGAGACAG
	mgfp	mgfp_F	ATGAGTAAAGGAGAAGAACTT	mgfp_R	CTTCTTAATTGTGTCAGCAACTTAATGATGGTGATGATGGTG
pSPAofaeB-mgfp	*SPAofaeB*	SPAofaeB_F	taccgaagaacgcaGCGGCCGCATGAAGGTCTCGCTGTGGCT	SSAofaeB_R	AAGTTCTTCTCCTTTACTCATGGCCAAGGCGAGAGATAGAT
	mgfp	mgfp_F	ATGAGTAAAGGAGAAGAACTT	mgfp_R	CTTCTTAATTGTGTCAGCAACTTAATGATGGTGATGATGGTG
pSPAomreA-mgfp	*SPAomreA*	SPAomreA_F	taccgaagaacgcaGCGGCCGCATGCCATCATTAAGCACCC	SSAomreA_R	AAGTTCTTCTCCTTTACTCATTGCCTCAATCAAGGGGGCAATG
	mgfp	mgfp_F	ATGAGTAAAGGAGAAGAACTT	mgfp_R	CTTCTTAATTGTGTCAGCAACTTAATGATGGTGATGATGGTG
pSPAopep-mgfp	*SPAopep*	SPAopep_F	taccgaagaacgcaGCGGCCGCATGCAGTTCCTCCCACCC	SSAopep_R	AAGTTCTTCTCCTTTACTCATCGCGCGGCTGAGCGAGGG
	mgfp	mgfp_F	ATGAGTAAAGGAGAAGAACTT	mgfp_R	CTTCTTAATTGTGTCAGCAACTTAATGATGGTGATGATGGTG
pSPAoalp1-mgfp +Aobet1	*PAoubi*	PAoubi_F	TCATTTACTCCCGAGGCGATCGCCGCCGGCGGGTCGCCAGAGTCAGGACACAG	PAoubi_R	GATGACGACGGTGAAAGTATTTCAGTTGTATAGAAGGTAG
	Aobet1	Aobet1_F	AATACTTTCACCGTCGTCATCATGGCTTCACGATTTCCACGCT	Aobet1_R	ctcaagttctgtagaatcaactacTCAAGTCAACCACACATAAGCA
	TAotef1	TAotef1_F	gtagttgattctacagaacttgagtcatgattttattcg	TAotef1_R	atcatctcccaaggaccgattgggatccaacacacaaactagta
pSPAoalp1-mgfp +Aosso1	*PAoubi*	PAoubi_F	TCATTTACTCCCGAGGCGATCGCCGCCGGCGGGTCGCCAGAGTCAGGACACAG	PAoubi_R	GATGACGACGGTGAAAGTATTTCAGTTGTATAGAAGGTAG

Plasmid	Amplified fragment	Forward primer	Sequence (5′–3′)	Reverse primer	Sequence (5′–3′)
	Aosso1	Aosso1_F	AATACTTTCACCGTCGTCATCATGAGTGGCGGCTACGGCTCATAC	Aosso1_R	ctcaagttctgtagaatcaactacTCACTTGTTTGCTTGGGTGAC
	TAotef1	TAotef1_F	gtagttgattctacagaacttgagtcatgattttattcg	TAotef1_R	atcatctcccaaggaccgattgggatccaacacacaaactagta
pSPAoalp1-mgfp+Aosly1	*PAoubi*	PAoubi_F	TCATTTACTCCCGAGGCGATCGCCGCCGGCGGGTCGCCAGAGTCAGGACACAG	PAoubi_R	GATGACGACGGTGAAAGTATTTCAGTTGTATAGAAGGTAG
	Aosly1	Aosly1_F	AATACTTTCACCGTCGTCATCATGGCGTCGCACTCAATGTCTC	Aosly1_R	ctcaagttctgtagaatcaactacTCAGCTTTCATGGCCAAGTTTAG
	TAotef1	TAotef1_F	gtagttgattctacagaacttgagtcatgattttattcg	TAotef1_R	atcatctcccaaggaccgattgggatccaacacacaaactagta
pSPAoalp1-mgfp+Aosec1	*PAoubi*	PAoubi_F	TCATTTACTCCCGAGGCGATCGCCGCCGGCGGGTCGCCAGAGTCAGGACACAG	PAoubi_R	GATGACGACGGTGAAAGTATTTCAGTTGTATAGAAGGTAG
	Aosec1	Aosec1_F	AATACTTTCACCGTCGTCATCATGAACTCCTCCATACTTAATATTC	Aosec1_R	ctcaagttctgtagaatcaactacCTATGACCGCCTAAAGAAGTGATg
	TAotef1	TAotef1_F	gtagttgattctacagaacttgagtcatgattttattcg	TAotef1_R	atcatctcccaaggaccgattgggatccaacacacaaactagta
pAocsn3	*Aocsn3*	Aocsn3_F	taccgaagaacgcaGCGGCCGCATGATGAAGTCCTTCTTCCTG	Aocsn3_R	CTTCTTAATTGTGTCAGCAACTTAGTGATGGTGATGGTGATGATG
pSPAoalp1-Aocsn3	*SPAoalp1*	SPAoalp1_F	taccgaagaacgcaGCGGCCGCATGCAGTCCATCAAGCGTAC	Aocsn3-SSAoalp1_R	GACCAGGAAGAAGGACTTCATCATGGCACCGAGGACCGCGGGA
	Aocsn3	SPAoalp1-Aocsn3_F	ATGATGAAGTCCTTCTTCCTGGTCGTGACCATCCTCG	Aocsn3_R	CTTCTTAATTGTGTCAGCAACTTAAACAGCAGTAGAGGTGACCTG
pSPAoalp1-Aocsn3+Aosly1	*PAoubi*	PAoubi_F	TCATTTACTCCCGAGGCGATCGCCGCCGGCGGGTCGCCAGAGTCAGGACACAG	PAoubi_R	GATGACGACGGTGAAAGTATTTCAGTTGTATAGAAGGTAG
	Aosly1	Aosly1_F	AATACTTTCACCGTCGTCATCATGGCGTCGCACTCAATGTCTC	Aosly1_R	ctcaagttctgtagaatcaactacTCAGCTTTCATGGCCAAGTTTAG
	TAotef1	TAotef1_F	gtagttgattctacagaacttgagtcatgattttattcg	TAotef1_R	atcatctcccaaggaccgattgggatccaacacacaaactagta
pSPAoalp1-AopafB+Aosly1	*SPAoalp1*	SPAoalp1_F	taccgaagaacgcaGCGGCCGCATGCAGTCCATCAAGCGTAC	AopafB-SSAoalp1_R	GACCAGGAAGAAGGACTTCATCATGGCACCGAGGACCGCGGGA
	AopafB	AopafB_F	ATGCATATCACCTCCATCGCCATCGTGTTCTTCGCCGCTATG	AopafB_R	CTTCTTAATTGTGTCAGCAACTCAGGGGGTCTGACAATCAACAC
	PAoubi	PAoubi_F	TCATTTACTCCCGAGGCGATCGCCGCCG	PAoubi_R	GATGACGACGGTGAAAGTATTTCAGTTGTATAGAAGGTAG

Plasmid	Amplified fragment	Forward primer	Sequence (5′–3′)	Reverse primer	Sequence (5′–3′)
			GCGGGTCGCCAGAG TCAGGACACAG		
	Aosly1	Aosly1_F	AATACTTTCACCGT CGTCATCATGGCGT CGCACTCAATGTCT C	Aosly1_R	ctcaagttctgtagaatcaactacTC AGCTTTCATGGCCAAG TTTAG
	TAotef1	TAotef1_F	gtagttgattctacagaacttgag tcatgattttattcg	TAotef1_R	atcatctcccaaggaccgattgggat ccaacacacaaactagta

Note: Lowercase letters indicate overlapping regions used for homologous recombination or yeast assembly.

Table S2. Oligonucleotide primers used for gene expression analysis

Gene	Amplicon size (kb)	Forward primer	Sequence (5′–3′)	Reverse primer	Sequence (5′–3′)
Aosly1	0.19	Aosly1_ RT_F	GGTCTATTCAGTGTT AGCGTC	Aosly1_ RT_R	CAGCACAGGTCGTGA CGTG
Aocsn3	0.24	Aocsn3_ RT_F	TACTATCAGCAGAAG CCAGTC	Aocsn3_ RT_R	TGATGGTTGGGATCTC AGTC
AopafB	0.37	AopafB_ RT_F	ATGCATATCACCTCC ATCGCCATC	AopafB_ RT_R	GACGGGGGTCTGACA ATCAACA
18S rRNA	0.18	18S rRNA_ RT_F	GTAACCCGTTGAACC CCATT	18S rRNA_ RT_R	CCATCCAATCGGTAGT AGCG

Note: *18S rRNA* was used as an internal reference gene for normalization of gene expression.

Table S3. Aoκ-casein titers quantified by ELISA, total extracellular protein and Aoκ-casein:total protein ratios from the recombinant strain grown in 4% SM medium or with increasing concentrations of yeast extract (YE)

Culture medium or YE concentration (g/L)	Total extracellular protein (mg/L)	Aoκ-casein (mg/L)	Aoκ-casein:total extracellular protein ratio
4% SM	5.4 ± 0.59	0.27 ± 0.01	0.049
5	8.73 ± 0.59	0.43 ± 0.08	0.049
10	34.49 ± 5.29	4.14 ± 0.12	0.120
15	91.01 ± 21.74	3.02 ± 0.08	0.033
20	97.24 ± 11.17	2.45 ± 0.1	0.025

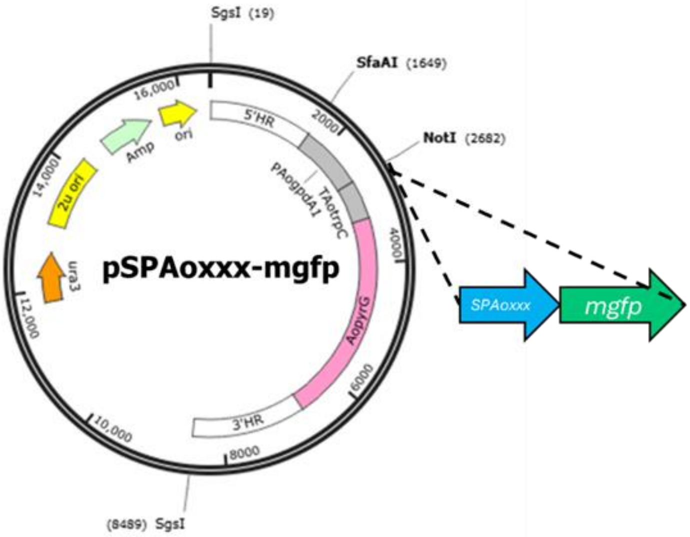

Figure S1. Plasmid map of pSPAoxxx-mgfp used for functional analysis of signal peptides

The construct contains an endogenous *Aspergillus oryzae* signal peptide sequence (*SPAoxxx*; blue arrow) fused in-frame to the monomeric green fluorescent protein reporter gene (*mgfp*; green arrow) under the control of the constitutive PAogpdA1 promoter. The plasmid backbone includes the *pyrG* selectable marker, homologous recombination regions, and bacterial propagation elements for cloning and maintenance.

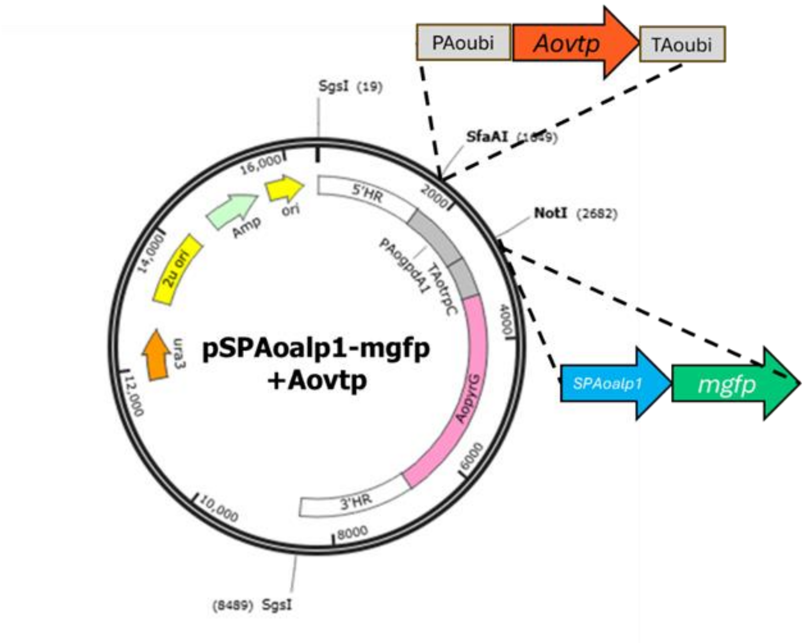

Figure S2. Plasmid map of pSPAoalp1-mgfp+Aovtp used for functional analysis of vesicle trafficking proteins

The construct contains the *Aspergillus oryzae* oryzin signal peptide (*SPAoalp1*; blue arrow) fused in-frame to the monomeric green fluorescent protein reporter gene (*mgfp*; green arrow). The vesicle trafficking protein gene (*Aovtp*; orange arrow) is expressed under the control of the *A. oryzae* ubiquitin promoter (PAoubi) and terminator (TAoubi). The plasmid backbone includes the *pyrG* selectable marker, homologous recombination regions, and bacterial propagation elements.

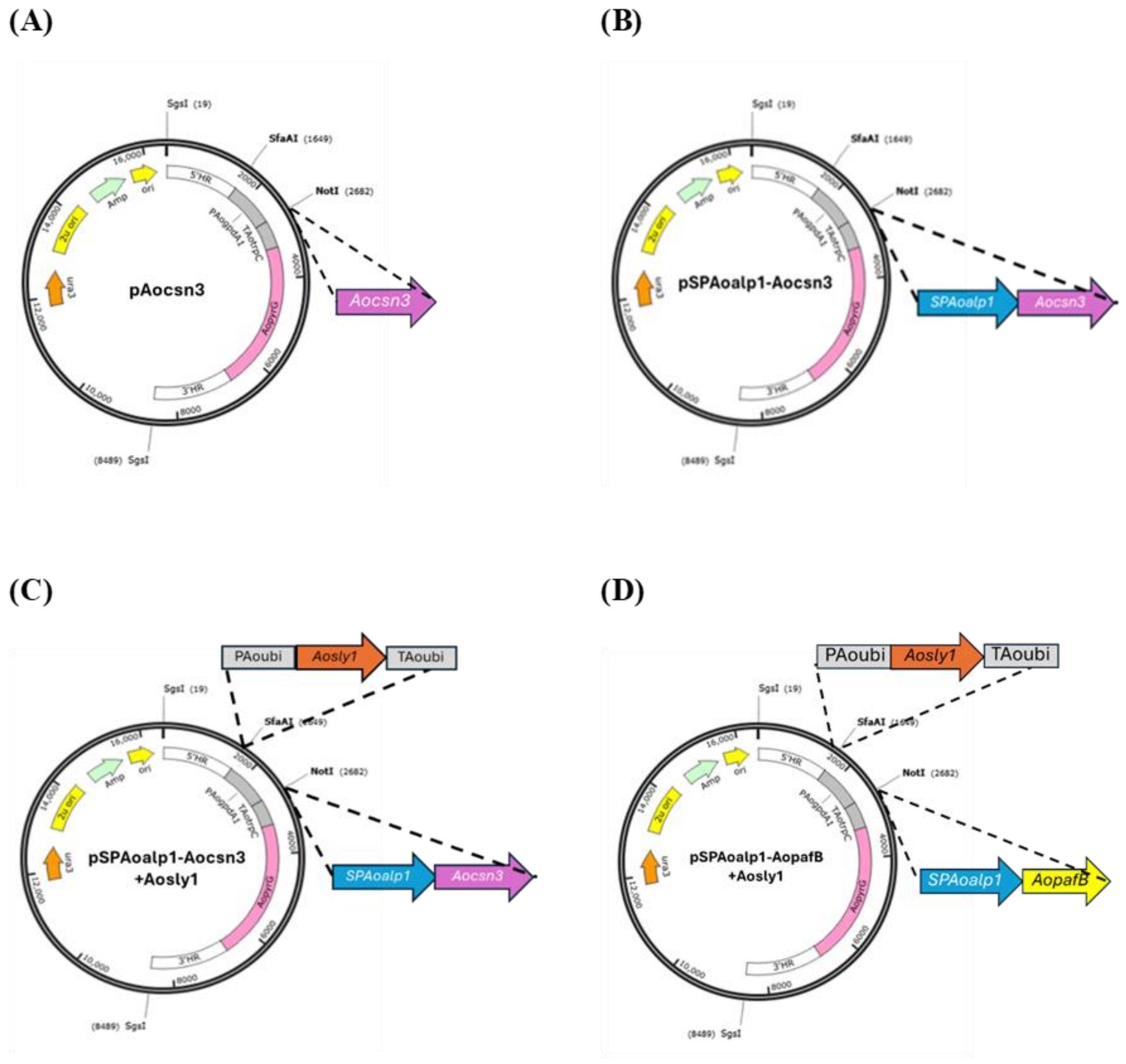

Figure S3. Plasmid maps used for heterologous protein expression in *Aspergillus oryzae*

(A) pAocsn3, expressing bovine κ-casein (*Aocsn3*; pink arrow) without the oryzin signal peptide.

(B) pSPAoalp1-Aocsn3, expressing κ-casein fused to the oryzin signal peptide (*SPAoalp1*; blue arrow).

(C) pSPAoalp1-Aocsn3+Aosly1, co-expressing *SPAoalp1-Aocsn3* with the Sec1/Munc18 family vesicle trafficking regulator Aosly1 (*Aosly1*; orange arrow).

(D) pSPAoalp1-AopafB+Aosly1, expressing the antifungal protein B (*AopafB*; yellow arrow) fused to *SPAoalp1* and co-expressed with *Aosly1*.

In all constructs, PAoubi and TAoubi denote the *A. oryzae* ubiquitin promoter and terminator, respectively (grey boxes). All genes and regulatory elements are derived from *A. oryzae* unless otherwise indicated.

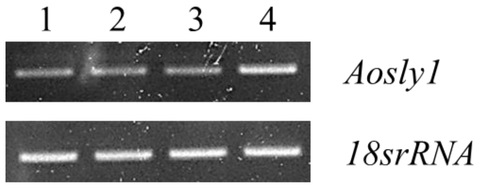

Figure S4. Reverse transcription polymerase chain reaction analysis of *Aosly1* expression in recombinant *Aspergillus oryzae* strains

Expression of *Aosly1* gene (0.19-kb) was investigated in wild-type (lane 1), no SP-GFP (lane 2), SPAoalp1-GFP (lane 3) and SPAoalp1-GFP+Aosly1 (lane 4) strains (top panel). Amplification of *18S rRNA* (0.18 kb) served as an internal housekeeping control (bottom panel).

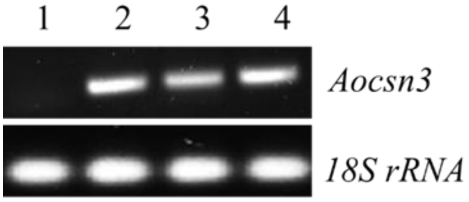

Figure S5. Reverse transcription polymerase chain reaction analysis of *Aocsn3* gene expression in recombinant *Aspergillus oryzae* strains

Expression of *Aocsn3* gene (0.24-kb) was examined in wild type (lane 1), Aoκ-casein (lane 2), SPAoalp1-Aoκ-casein (lane 3), and SPAoalp1-Aoκ-casein+Aosly1 (lane 4) strains (top panel). Amplification of *18S rRNA* (0.18 kb) served as an internal housekeeping control (bottom panel).

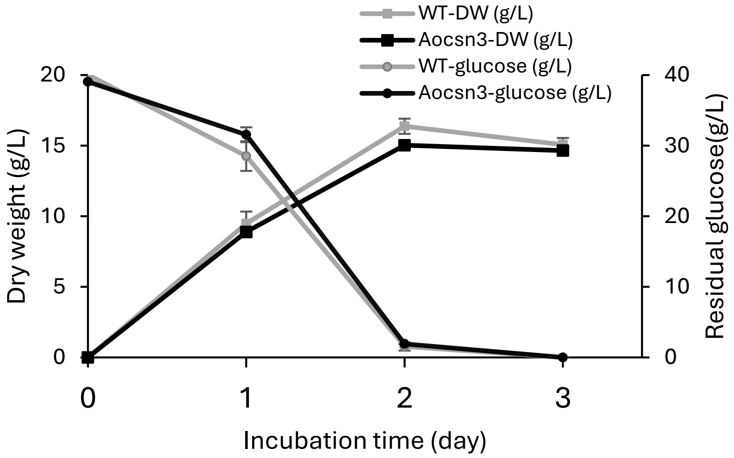

Figure S6. Growth characteristics of the recombinant Aoκ-casein strain

Mycelial growth (dry weight; DW) and residual glucose profiles of the recombinant strain (black line) and the wild-type strain (WT) (grey line) in the basal semi-synthetic medium broth at 30 °C and 200 rpm for 3 d. Symbols indicate DW (squares) and residual glucose concentration (dots). The data are represented as the mean value with standard deviation (mean ± SD)

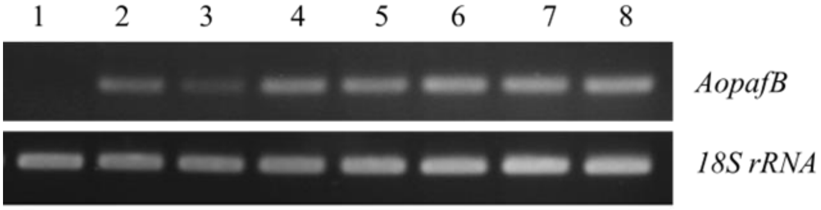

Figure S7. Reverse transcription polymerase chain reaction analysis of *AopafB* gene expression in recombinant *Aspergillus oryzae* strains

Expression of the *AopafB* gene (0.37-kb) was examined in the wild type strain (lane 1) and in SPAoalp1-AopafB+Aosly1 transformant clones 1−7 (lanes 2–8) (top panel). Amplification of *18S rRNA* (0.18 kb) served as an internal housekeeping control (bottom panel).

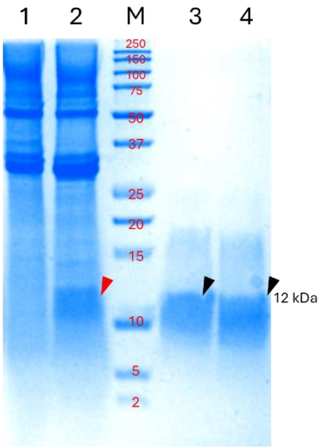

Figure S8. Analysis of the ~12 kDa band detected in the supernatant from the AoPAFB-expressing strain using a Tris-tricine-SDS buffer system with strongly reducing conditions

Analysis of the secretion derived from concentrated supernatant samples of wild-type (lane 1) and AoPAFB-expressing strain (lane 2). A ~12 kDa band (putative dimer) detected in recombinant strain is marked by red arrowhead; prestained protein molecular weight marker (lane M); synthetic PAFB standard (~12 kDa, putative dimer) with and without dithiothreitol (DTT) treatment (lanes 3 and 4 respectively), indicated by black arrowheads. Each lane was loaded with 50 µg of total protein.
